# Mechanistic modeling of insecticide risks to breeding birds in North American agroecosystems

**DOI:** 10.1371/journal.pone.0176998

**Published:** 2017-05-03

**Authors:** Matthew Etterson, Kristina Garber, Edward Odenkirchen

**Affiliations:** 1 USEPA Office of Research and Development, National Health and Environmental Effects Research Laboratory, Mid-Continent Ecology Division, Duluth, Minnesota, United States of America; 2 USEPA Office of Pesticide Programs, Environmental Fate and Effects Division, Washington, DC, United States of America; Universiteit Gent, BELGIUM

## Abstract

Insecticide usage in the United States is ubiquitous in urban, suburban, and rural environments. There is accumulating evidence that insecticides adversely affect non-target wildlife species, including birds, causing mortality, reproductive impairment, and indirect effects through loss of prey base, and the type and magnitude of such effects differs by chemical class, or mode of action. In evaluating data for an insecticide registration application and for registration review, scientists at the United States Environmental Protection Agency (USEPA) assess the fate of the insecticide and the risk the insecticide poses to the environment and non-target wildlife. Current USEPA risk assessments for pesticides generally rely on endpoints from laboratory based toxicity studies focused on groups of individuals and do not directly assess population-level endpoints. In this paper, we present a mechanistic model, which allows risk assessors to estimate the effects of insecticide exposure on the survival and seasonal productivity of birds known to forage in agricultural fields during their breeding season. This model relies on individual-based toxicity data and translates effects into endpoints meaningful at the population level (i.e., magnitude of mortality and reproductive impairment). The model was created from two existing USEPA avian risk assessment models, the Terrestrial Investigation Model (TIM v.3.0) and the Markov Chain Nest Productivity model (MCnest). The integrated TIM/MCnest model was used to assess the relative risk of 12 insecticides applied via aerial spray to control corn pests on a suite of 31 avian species known to forage in cornfields in agroecosystems of the Midwest, USA. We found extensive differences in risk to birds among insecticides, with chlorpyrifos and malathion (organophosphates) generally posing the greatest risk, and bifenthrin and λ-cyhalothrin (pyrethroids) posing the least risk. Comparative sensitivity analysis across the 31 species showed that ecological trait parameters related to the timing of breeding and reproductive output per nest attempt offered the greatest explanatory power for predicting the magnitude of risk. An important advantage of TIM/MCnest is that it allows risk assessors to rationally combine both acute (lethal) and chronic (reproductive) effects into a single unified measure of risk.

## Introduction

As environmental contaminants, insecticides may pose concerns because they are distributed intentionally and are designed to affect their targeted pests. They may also impact non-targeted organisms. Pesticide exposure may adversely affect a wide range of terrestrial animals including beneficial arthropods [1], mammals [2] and birds [2, 3], causing both mortality [4] and sublethal reproductive effects [5]. Indirect effects on birds have also been reported from insecticide-caused reductions in their invertebrate prey base [6]. Current insecticides act through varying modes of action. For example, organophosphates and carbamates inhibit acetylcholinesterase, leading to overstimulation of the nervous system and death [7] and these pesticides have been implicated in numerous wildlife poisonings [8, 9]. Insectivorous birds may be particularly susceptible because modes of action are conserved across animal taxa and many avian species are known to forage in agricultural fields [10].

Over the last century, the amount of conventional pesticide mass applied overall in the U.S. has generally increased to current use levels of around 1.2 billion lbs active ingredient (a.i.) per year by the 1990s [11]. In 2006–2007, estimated annual use of pesticide a.i.’s in the U.S. was 1.1 billion pounds; with approximately 90–100 million of those pounds represented by insecticides [12]. The set of specific a.i.’s used at any given time is dynamic, changing in response to many factors, including changing crops, pest pressure, changing economics of pest control, development of new insecticides, evolution of resistance, and increased understanding of risks associated with specific products [11]. Ecological risk assessments for insecticides are typically evaluated on an insecticide-by-insecticide basis (i.e., a single a.i. at a time) for registration of a new insecticide. As prescribed in the Federal Insecticide, Fungicide, and Rodenticide Act (FIFRA) (7 U.S.C. §§136-136y), insecticides and other pesticides are also re-evaluated every 15 years. Risk assessments for single a.i.’s are difficult to interpret in isolation because mitigation and risk management for a given insecticide may result in increased usage of other registered insecticides labeled for use on the same crop, which carry their own set of risks. FIFRA also mandates that the U.S. Environmental Protection Agency (USEPA) consider the economic benefit of insecticides as well as their ecological risks when making risk management decisions.

In evaluating data for an insecticide registration application and for registration review, scientists from the USEPA Office of Pesticide Programs (OPP) follow a process referred to as ecological risk assessment (ERA) [13], which includes three general steps: problem formulation, analysis, and risk characterization. These steps may be repeated in iterative tiers along a continuum of decreasing generality and increasing realism, halting if a lower-tier assessment suggests little or no ecological risk. At each tier, EPA scientists assess the fate of the insecticide and the risks the insecticide poses to the environment. Models are used in each tier and, like the risk assessment, move from general (tier 1) to realistic (tier 3) as greater precision and understanding of risk is required. These risk assessments consider major transport pathways (*e*.*g*., volatilization, spray drift) from sites where the insecticide is applied and degradation using data submitted by companies supporting registrations for insecticide a.i.’s and their formulated products. Exposures via relevant routes (*e*.*g*., food, dermal contact, drinking water, inhalation) are assessed using simulation models and empirical measures in the environment. The risks associated with those estimated exposures are evaluated using available toxicity data that define thresholds for mortality, growth, reproduction, and other sublethal effects that are relevant to the individual or group of individuals.

For birds, the first tier of ERA starts with the T-REX model [14], which provides a conservative estimate of exposure through diet. Tier I assessments are based on risk quotients (RQs), which are calculated by dividing a conservative estimate of exposure by a threshold toxicity value representing mortality and sublethal effects (*e*.*g*., the median lethal dose or LD_50_; the median lethal concentration LC_50_; the No Observed Adverse Effect Concentration or NOAEC; the No Observed Adverse Effect Level or NOAEL). After RQs are calculated, they are compared to levels of concern (LOCs) in order to determine whether an insecticide use poses risks of concern for mortality or reproductive effects to birds through dietary exposure. For RQs indicating potential acute effects (exposures compared to the adult LD_50_ or juvenile LC_50_), the (acute) LOC is 0.5 (for species not federally listed as threatened or endangered under the Endangered Species Act). For RQs indicating potential non-lethal adverse effects (exposure exceeding the NOAECs from the reproduction test), the (chronic) LOC is 1.0. If RQs are below the acute and chronic LOCs, it is concluded that the insecticide does not pose a risk of concern to birds. In cases where an RQ exceeds an LOC, there is potential risk of effects, and a higher tier assessment may be warranted. For higher tier avian risk assessments, OPP uses the Terrestrial Investigation Model (TIM, v.3.0) [15], a probabilistic model that focuses on acute exposures to birds. For chronic risks, OPP uses the Markov Chain Nest Productivity Model (MCnest) [16, 17], a mechanistic model of avian breeding, to assess potential declines in the annual reproductive success of exposed bird populations.

Applications for pesticide registration in the US include specific proposals, referred to as the formulated product label, for crops on which the product would be used, and relevant application information (e.g., maximum application rates, application methods, number of applications per year). Typically, pesticides will have multiple registered labels for different crops and different regions of the US. Thus labeling is one of the major tools used by USEPA to limit human and ecological risk in different societal and ecological contexts. Insecticide risk assessments at USEPA consider the labeled application rates for specific pests of crops or groups of crops and the non-target organisms that may be exposed through those applications.

The risk assessment described in this paper is focused on some representative insecticides registered for use on corn. Corn is the most widely planted crop in the United States, with approximately 35 million hectares (> 87 million acres) planted in 2012 [18]. At least 68 bird species have been documented to use corn fields and adjacent habitats during the typical northern temperate breeding season [10,15,19,20] and are thus potentially exposed to insecticides used for pest control on corn. Some of the major pests on this crop include corn root worm borer, cutworm, and armyworm [21, 22]. Armyworm (*Pseudaletia unipuncta*), a native species distributed widely in the Eastern U.S., feeds on leaves of grasses and damages corn crops through defoliation. Cutworms of a variety of species, including black cutworm (*Agrotis ipsilon*), another native species of the eastern U.S., feed on young corn sprouts, typically cutting them off at emergence from the soil. Finally, corn borers, such as the European corn borer (*Ostrinia nubilalis*), an introduced pest, bore tunnels in cornstalks, weakening and toppling the plants as they grow taller and heavier. Given that these three pests target different lifestages of corn and have different phenologies of crop damage, optimal timing for control of these pests via insecticide application varies [22]. Many different insecticides are registered for use on corn to control these pests, including organophosphates, carbamates, pyrethroids, neonicotinoids, and diamides.

Below, the integrated TIM/MCnest model for avian risk assessment for insecticides is introduced and applied to a relative risk assessment for one growing season for several commonly used insecticides from different chemical classes and modes of action. The purpose of TIM/MCnest is to provide risk assessors at USEPA with a mechanistic tool for evaluating the risk of adverse effects to bird populations exposed to pesticides during the breeding season. Specific objectives of this evaluation include the following: 1) to introduce and describe the new integrated TIM/MCnest model and demonstrate how it can integrate acute and chronic effects for birds into a single assessment endpoint (reduction in seasonal fecundity); 2) to show how TIM/MCnest can be applied to evaluate the relative risk of insecticides in agroecosystems; 3) to evaluate the relative risk to birds using cornfields of 12 commonly used pesticides; and 4) to examine the avian ecological characteristics that are predictive of greater relative risk of adverse effects.

## Methods

### Models and their integration

TIM v.3.0 (hereafter referred to as TIM) [15] is an exposure and effects model that can be used to predict avian mortality from acute insecticide exposure resulting from a realistic time-dependent pesticide use scenario. This time frame corresponds to one growing season of the treated crop or a single sub-annual insecticide application window. The spatial scale is at the field level; however, specific field dimensions are undefined. It is assumed that the field and surrounding area meet habitat and dietary requirements for the modeled species. During the simulation, birds use the treated field and edge habitat to meet their requirements for food and water. TIM also accounts for exposure via dermal and inhalation routes for birds on the field or for adjacent habitat that receives spray drift. It is expected that the relative importance of these routes of exposure will vary based on the properties of the insecticide, its labeling and use patterns, as well as the characteristics of the simulated bird species. Risk, expressed as a function of exposure (dose) and toxicity, is assessed for liquid spray applications of an insecticide made to vegetation or bare ground in the field. Insecticide application methods that may be modeled in TIM include: aerial, airblast, ground broadcast, ground banded, and ground in furrow. For all of these application methods, exposure can be assessed on the treated field and edge habitat where spray drift is transported. Major assumptions of TIM include:

1. Toxicity of simulated birds is represented by surrogate test species;
2. Dose is a function of diet, inhalation, drinking water and dermal uptake, as well as elimination;
3. Intake rates are allometrically scaled;
4. Birds move on and off of treated fields;
5. Birds follow a bimodial feeding pattern (where peak feeding occurs soon after sunrise and before sunset);
6. Residues on insects represent values present on the 90th percentile of agricultural fields;
7. Acute toxicity (LD50) is scaled using body weight and, where available, empirical insecticide-specific scaling factors [23].

The Markov chain nest productivity model (MCnest) is a mechanistic model that estimates declines in reproductive success for temperate zone land birds during the breeding season [16,17]. The model uses a Markovian transition matrix [24] to follow a standard algorithm [25] of nest establishment, survival, failure, and re-nesting up to the end of a typical breeding season. The number of successful nests per breeding female in a single breeding season is tracked, along with the cause for each failed nest. Insecticide induced failures are modeled by the inclusion of an exposure algorithm, which generates daily exposure values. These values are compared to phase-specific NOAELs for different kinds of adverse effects that might be induced by insecticide exposure (*e*.*g*., eggshell thinning, reduced egg viability, reduced hatching success, increased abandonment) [16]. Populations with identical parameter values are simulated with replication to provide estimates of variability around model predictions. MCnest output consists of estimates of the expected number of successful young per female in a population exposed to a given insecticide use scenario. Bennett and Etterson [26–28] provide detailed information on MCnest, including its use, technical background, and species library. Important assumptions underlying MCnest are:

1. Females necessarily attempt to renest if there is sufficient time remaining in the breeding season;
2. Demographic parameters, such as nest survival, clutch size, and waiting periods post failure and post fledging are fixed;
3. Nest failures occur as a unit (i.e., not on a per-egg basis), whether natural or induced by pesticide exposure;
4. Exceedance of phase-specific toxicity endpoints results in complete nest failure;
5. NOAELs from the avian reproduction test are generally applicable across species.

**Fig 1:**
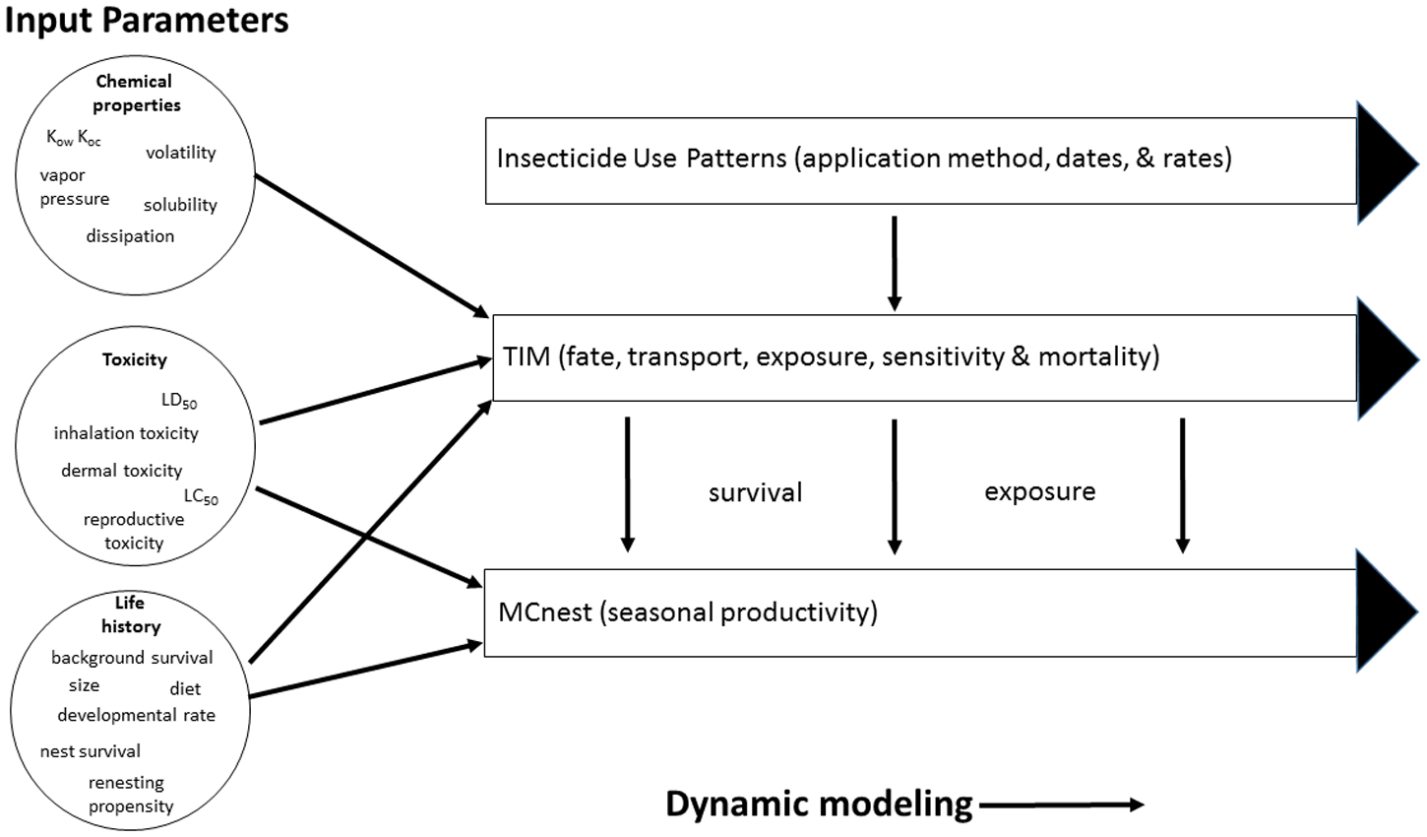
Conceptual diagram of the integration of TIM and MCnest. Circles show qualitative groups of data inputs with (non-exhaustive) examples of types of data in each group. Arcs illustrate data inputs to each model. Rectangles with arrows illustrate dynamic models running over the course of an avian breeding season in response to (user-designed) specific insecticide use patterns.

TIM and MCnest were integrated within the Matlab [29] programming environment by having MCnest call TIM as a subroutine. To achieve the merger of the two models, TIM, which uses an hourly time step, was modified to produce daily summaries of exposure for both adults and juveniles. Similarly, MCnest was modified to include adult mortality, as predicted by TIM. The simulation first runs TIM on adults to generate exposure and mortality results for each simulated breeding female; reproduction is then simulated using MCnest conditional on the mortality and exposure profiles generated from TIM. Thus, in the merged TIM/MCnest model, exposure and adult mortality is estimated by TIM, and reproductive effects are estimated by MCnest, conditional on the exposure and mortality estimates produced by TIM (Fig 1). A beta version of the integrated model is available online at: http://www.epa.gov/endangered-species/provisional-models-endangered-species-pesticide-assessments. Additional important assumptions made for the integrated model include:

1. The field and surrounding area meet habitat and dietary requirements for the modeled species;
2. When birds finish breeding they disperse from the treated field and thus receive no further exposure.

### Avian species

To determine which bird species to simulate we compiled a list of 68 species observed using agricultural fields in Iowa and Illinois, based on standardized survey methods [10,15,19,20]. For example, Best et al. [10] used transects at the center and edges of cornfields to record all species seen or heard. Transects were conducted between dawn and 9 am, 3 times weekly, between 9 May and 9 July during the avian breeding season. These studies documented the number of individuals within a species that were observed in corn fields and their adjacent habitats (*e*.*g*., forest, grassland). The intersection of this list and the existing MCnest library [28] was then taken, which resulted in 31 species with the required demographic information and which have been observed on Midwestern cornfields during the breeding season (Table 1). Sources for life-history data for these species are provided in the MCnest species library [28] and life-history data are provided in S1 Appendix.

**Table 1:** Species modeled.

Common Name	Scientific Name
Canada goose	*Branta canadensis*
northern bobwhite	*Colinus virginianus*
killdeer	*Charadrius vociferus*
mourning dove	*Zenaida macroura*
northern flicker	*Colaptes auratus*
eastern phoebe	*Sayornis phoebe*
eastern kingbird	*Tyrannus tyrannus*
blue Jay	*Cyanocitta cristata*
American crow	*Corvus brachyrhynchos*
horned lark	*Eremophila alpestris*
barn swallow	*Hirundo rustica*
black-capped chickadee	*Poecile atricapillus*
house wren	*Troglodytes aedon*
Eastern bluebird	*Sialia sialis*
American robin	*Turdus migratorius*
cedar waxwing	*Bombycilla cedrorum*
common yellowthroat	*Geothlypis trichas*
yellow warbler	*Setophaga petechia*
chipping sparrow	*Spizella passerina*
field sparrow	*Spizella pusilla*
vesper sparrow	*Pooecetes grammineus*
savannah sparrow	*Passerculus sandwichensis*
grasshopper sparrow	*Ammodramus savannarum*
Northern cardinal	*Cardinalis cardinalis*
dickcissel	*Spiza americana*
red-winged blackbird	*Agelaius phoeniceus*
eastern meadowlark	*Sturnella magna*
western meadowlark	*Sturnella neglecta*
common grackle	*Quiscalus quiscula*
American goldfinch	*Carduelis tristis*
house sparrow	*Passer domesticus*

### Insecticides & simulations

The effects of twelve current use insecticides to birds (Table 2) were modeled following current labeling guidelines for these products on corn in Illinois. These insecticides were selected because they are all registered for application to corn to control armyworms, cutworms and/or corn borers. In addition, many are recommended for use by extension agents (*e*.*g*., [30–32]). These insecticides represent seven distinct chemical classes with different modes of action on insect pests and on non-target organisms (*e*.*g*., birds). Among these 12 pesticides, the maximum number of applications allowed under the label ranged from 1 to 14. Maximum application rates ranged from 0.03 lbs/acre (λ-cyhalothrin) to 2 lbs/acre (carbaryl) (Table 2). This application information was used to define input values in model simulations. Representative product labels are also described in Table 2.

**Table 2:** Insecticides considered in this analysis. Application rates, numbers and intervals are based on representative product labels.

Pesticide	Class	Mode of Action	Representative product (registration #)	Application rate (Lb a.i./A)^1^	Number of applications (interval in days)^1^
bifenthrin	pyrethroid	Sodium channel modulator	Brigade 2 EC ^®^ (279–3313)	0.1	1 (NA)
carbaryl	carbamate	AChE inhibition	Sevin 4F ^®^ (61842–38)	2	4 (14)
chlorantraniliprole	diamide	Ryanodine receptor activator	Coragen ^®^ (352–729)	0.065	3 (7)
chlorpyrifos	organophosphate	AChE inhibition	Lorsban ^®^ 75WG (62719–301)	1	3 (10)
cyfluthrin	pyrethroid	Sodium channel modulator	Tombstone ^®^ (34704–912)	0.044	4 (7)
esfenvalerate	pyrethroid	Sodium channel modulator	Asana XL ^®^ (59639–209)	0.05	5 (3)
indoxacarb	oxadiazines	Voltage-dependent sodium channel blocker	Avaunt ^®^ (352–597)	0.065	4 (3)
λ-cyhalothrin	pyrethroid	Sodium channel modulator	Warrior II ^®^ (100–1295)	0.03	4 (3)
malathion	organophosphate	AChE inhibition	Malathion 5EC ^®^ (66330–220)	1	2 (7)
methomyl	carbamate	AChE inhibition	Lannate ^®^ (352–384)	0.45	14 (1)
methoxyfenozide	diacylhydrazine	ecdysone agonist	Intrepid 2F ^®^ (62719–442)	0.25	4 (5)
permethrin	pyrethroid	Sodium channel modulator	Permethrin 3.2 EWC ^®^ (53883–73)	0.24	6 (3)

^1^Applications made via foliar spray. All pesticides may be applied via ground or aerial methods, except for indoxacarb, which may only be applied by ground.

Risks of mortality and reproductive effects to birds were first assessed using the OPP Tier I risk assessment method (T-REX). Insecticide-specific input values used to run T-REX are provided in S2 Appendix. Insecticides that passed the Tier I assessment were not considered further.

Those insecticides with risks of concern for mortality (acute RQ > 0.5) or reproductive effects (chronic RQ > 1.0) were modeled using the TIM/MCnest integrated model for the 31 species described above (Table 1). TIM/MCnest simulations were run for flocks of 25 females with 400 replicates each (10,000 total birds simulated for each scenario). This level of replication was used in TIM to estimate the probability distribution of mortality to a group of 25 female birds breeding in proximity to a treated field, and was used in MCnest to obtain a distribution around expected seasonal fecundity. Each simulation was run for the duration of a full breeding season for the species simulated. When application dates fell outside the breeding season for a given species, the simulation duration was extended to include all application dates as well as the breeding season of the simulated species. Parameter values for all TIM and MCnest simulations are provided in the Supporting Information (S2 and S3 Appendices).

Pesticide applications were simulated by specifying a date of first application for all pesticides and then following the pesticide labels (Table 2) to determine the dates of subsequent applications. For example, for a first application date of 20 May, chlorpyrifos applications were simulated on 20 May, 30 May, and 9 June (3 applications separated by 10 days). For each pesticide and each first application date, we simulated maximum application rates and maximum (up to 5) numbers of applications, together with the minimum interval (or 7 days, whichever is longer) between applications (Table 2). Four first application dates 20 May, 20 June, 21 July, or 21 August were chosen to reflect the most likely timing for controlling corn borers, armyworms, and cutworms. This range of dates was established by considering label language relevant to the pests and crop stage, the susceptible life-stages of corn to a given pest, and the likely developmental stage of corn on a given date in our landscape [33]. For corn root worm borer and cutworm, applications are likely to be made in June. Insecticide applications may be made to control army worm in June or July. Because it tends to result in greater exposure to adjacent habitats, we conservatively modeled all applications as aerial spray for all insecticides, though one, indoxacarb, is not registered for aerial applications.

TIM and MCnest are currently designed to simulate foliar spray applications of insecticides. The model does not account for exposures due to seed treatments or granular formulations. Therefore, insecticides registered for use on corn that are only formulated as granules (*e*.*g*., terbufos) or seed treatments (*e*.*g*., clothianidin, thiamethoxam) were not considered in this analysis.

### Toxicity & physical chemistry

Toxicity data for the Tier 1 T-REX analysis and for TIM/MCnest modeling were drawn from standard toxicity tests required to support insecticide registration under FIFRA. The three most pertinent tests are the avian acute oral toxicity test [34], the avian dietary toxicity test [35], and the avian reproduction test [36] (Table 3). Additional data were drawn from rat toxicity studies and magnitude of residue studies for livestock (specifically chickens; [37]) conducted as part of human health risk assessment [38, 39]. Data required by TIM on the physical, chemical and fate properties (*e*.*g*., K_ow_, K_oc_, water solubility, Henry’s Law constants, vapor pressure) of insecticides are also required to support product registration or were calculated from submitted data. (See Supporting Information, S3 Appendix for full parameter sets for both models).

**Table 3:** Primary toxicity values for insecticides modeled using TIM/MCnest.

Pesticide	LD50^1^^,^^2^	LC50^1^^,^^3^	NOEC^1^^,^^3^	Reproductive effect (reduction in)
carbaryl	2000m	>5000q	>2930q	eggs laid
chlorpyrifos	29.2q	203m	39.2q	eggs laid
indoxacarb	98q	808q	144q	pre-laying weight
λ-cyhalothrin	5000m	2354q	4.62m	eggs laid
malathion	359q	3497q	358q	eggs laid, egg viability, eggshell thickness
methomyl	24.2q	3714m	153q	eggs laid
permethrin	11274m	>10,000q	>472q	none

^1^Tested species: m = mallard; q = northern bobwhite;

^2^mg a.i./kg-bodyweight;

^3^mg a.i./kg-diet

### Sensitivity analysis

To identify life-history characteristics that make species more vulnerable to insecticide effects in the TIM/MCnest model, we conducted a global sensitivity analysis between model predictions of seasonal fecundity and 13 model parameters for ecological characteristics that we thought might influence vulnerability to insecticide effects (Table 4). Because avian life-history characteristics are often highly correlated [40], we chose to evaluate global sensitivity using the life-history variation present in the 31 modeled species, rather than the more typical method of estimating local sensitivity by modifying a single parameter and rerunning the model to observe the effect on the response variable [41]. Thus our strategy followed the recommendation of Cariboni et al. [41] that an ideal sensitivity analysis should “operate simultaneously on all uncertain inputs,” where the scope of uncertainty of interest here was the life-history variation among species modeled; sensitivity to chemical properties was not assessed. We used the model results from the simulations described above and ran univariate linear regressions of predicted fecundity on each of 13 model parameters, using the 10,000 simulated females of the 31 species as replicates. We used R^2^ to rank the resulting regressions in terms of their explanatory power for predicted fecundity for the 31,000 simulated females. These regressions were run separately by month of first application (May, June, July, and August) and by insecticide.

**Table 4:** Parameters included in sensitivity analysis.

Parameter	Definition
body.weight	Species typical female body weight (g)
trophic	Species diet class (frugivore, granivore, herbivore, insectivore, omnivore)
egg.surv	Daily nest survival rate during incubation
nest.surv	Daily nest survival rate during nestling care (post-fledging care for precocial birds)
season.start	Serial date (since 1 January) on which breeding season begins
season.end	Serial date (since 1 January) on which breeding season ends
season.length	Length of breeding season (season.end—season.start)
FPSN	Mean number of fledglings per successful nest
frequency.on.field	Frequency of observation of species on agricultural fields (from census studies)
fidelity	Tendency for species to continue to forage at a site versus move to a new site
nest.cycle	Length of the nest cycle (days, first egg through fledging)
wait.fail	Typical duration, in days, between failure of nest and start of a new nest
wait.succ	Typical duration, in days, between successful nest and start of a new nest

## Results

### Insecticides

Of the 12 insecticides considered (Table 2), 5 passed the Tier I assessment conducted with T-REX (bifenthrin, chlorantraniliprole, cyfluthrin, esfenvalerate and methoxyfenozide) and were not considered further. Full results for the Tier I risk assessment are provided in S2 Appendix.

The remaining 7 insecticides (carbaryl, chlorpyrifos, indoxacarb, λ-cyhalothrin, malathion, methomyl, and permethrin) had either chronic or acute RQs greater than their corresponding level of concern (S2 Appendix). These 7 insecticides differed considerably in their chemical properties (S3 Appendix). For example, water solubility (mg a.i./L) ranged from 0.005 (λ-cyhalothrin) to 55,000 (methomyl), whereas the octanol water partitioning coefficient (K_ow_) ranged from 20.4 (methomyl) to over 10^6^ (λ-cyhalothrin and permethrin). Foliar dissipation half-life (days) varied from 2.5 (methomyl) to 22.5 (indoxacarb). Similar variation was observed among toxicity values. For example, the oral LD_50_ (mg a.i./k.g. body weight) ranged from 24.2 (methomyl) to >11,274 (permethrin), and the dietary concentrations (mg a.i./kg diet), corresponding to the NOAELs for reproduction endpoints, ranged from 4.62 (λ-cyhalothrin) to 2,930 (carbaryl). Thus, for these insecticides, acute and chronic toxicity varied over three orders of magnitude, and chemical properties that influence exposure varied over at least four orders of magnitude.

### Species

Among the 31 species modeled, all but 5 were in the order Passeriformes with one member each of Anseriformes, Galliformes, Charadriiformes, Columbiformes, and Piciformes (Table 1). Ecological traits also varied among the 31 species. Insectivores were the dominant trophic group (16 species) followed by 7 omnivores, 6 granivores, an herbivore, and a frugivore (Table 1, S1 Appendix). Body weight ranged from 9.6 g (yellow warbler) to 4.8 kg (Canada goose). Timing of the breeding season also varied considerably, with the earliest season (Canada goose) beginning on 20 March and ending on 4 April, and the latest season (American goldfinch) lasting from 1 July through 1 September. The duration of the breeding season ranged from 21 days (yellow warbler) to 168 days (mourning dove). This variation in life-history provides further potential for modifying risk of exposure and adverse effects among species.

### Model results

Simulated exposures showed differences in risk among insecticides and among species. These risks can be further partitioned among risk of lethal versus reproductive effects. Furthermore, the magnitude of effects differed among insecticides and by timing of application. Below we show a subset of results chosen to highlight important patterns of variation by ecological traits. Full results are reported in the Supporting Information (S4 Appendix).

**Fig 2:**
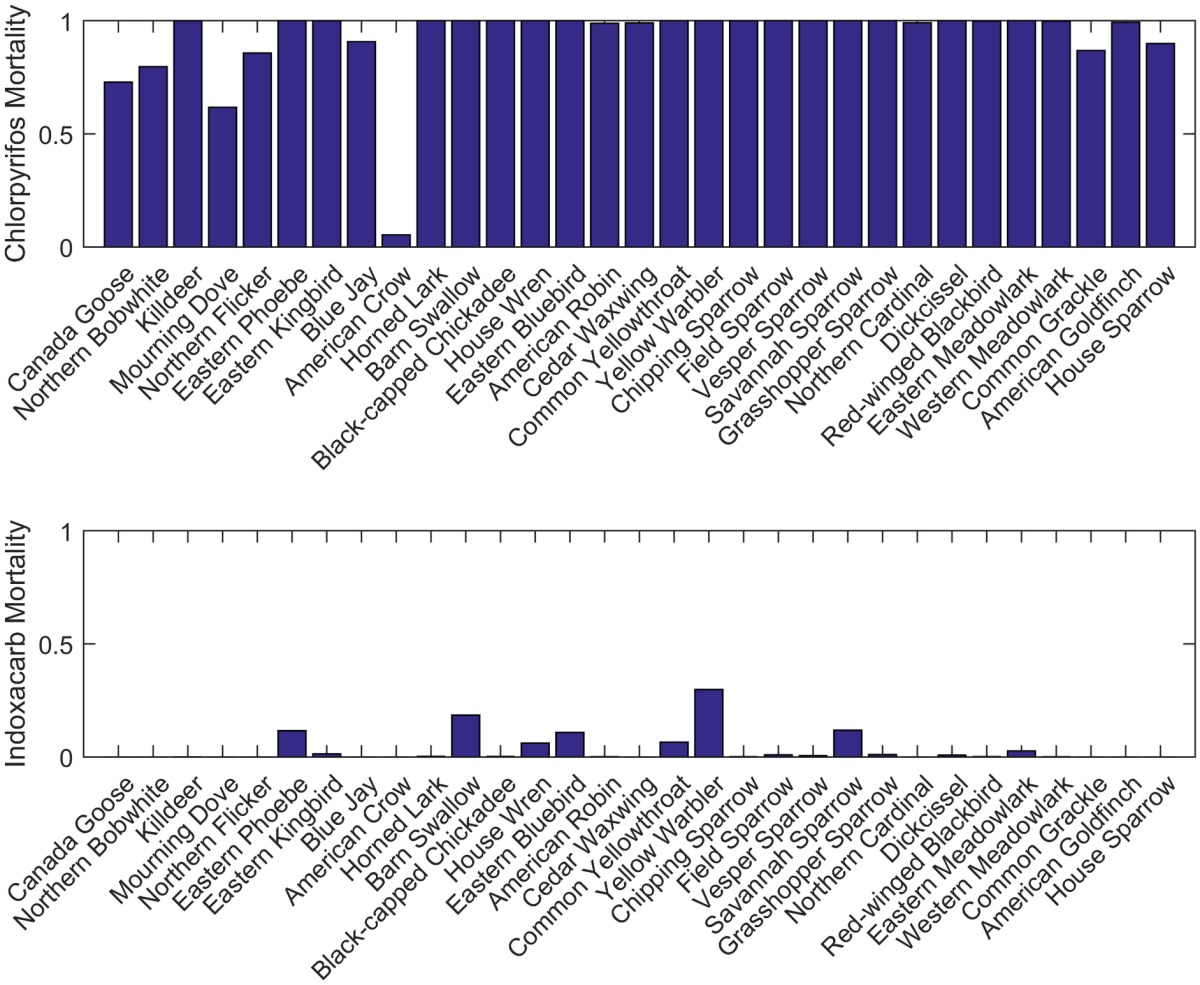
Comparison of predicted mortality between chlorpyrifos and indoxacarb. Vertical axis represents the model prediction for the proportional mortality to simulated birds.

Predicted mortality ranged from zero to 100% for different species and different insecticides (Tables A-G, Fig A in S4 Appendix). In general, the acetylcholinesterase inhibitors (excepting carbaryl, Tables B, E, F in S4 Appendix) showed high risk of mortality, whereas the remaining three insecticides and carbaryl showed moderate to low mortality (Tables A, C, D, G in S4 Appendix). Fig 2 contrasts expected mortality for chlorpyrifos and indoxacarb. Although there are clear differences among insecticides in risk of mortality, for a given insecticide mortality rates also differed among species (Fig 2). Although sample size was small for these groups, herbivores, omnivores, and the frugivore seemed to show different patterns of mortality than insectivores, due to higher predicted residues on invertebrate prey than on other dietary items. Mortality patterns were fairly consistent across dates, with the exception of the August application for which mortality was much lower for most species. This result is due to many species having finished breeding by 20 August, at which point we assume that they have dispersed from the treated field and thus receive no exposure.

**Fig 3:**
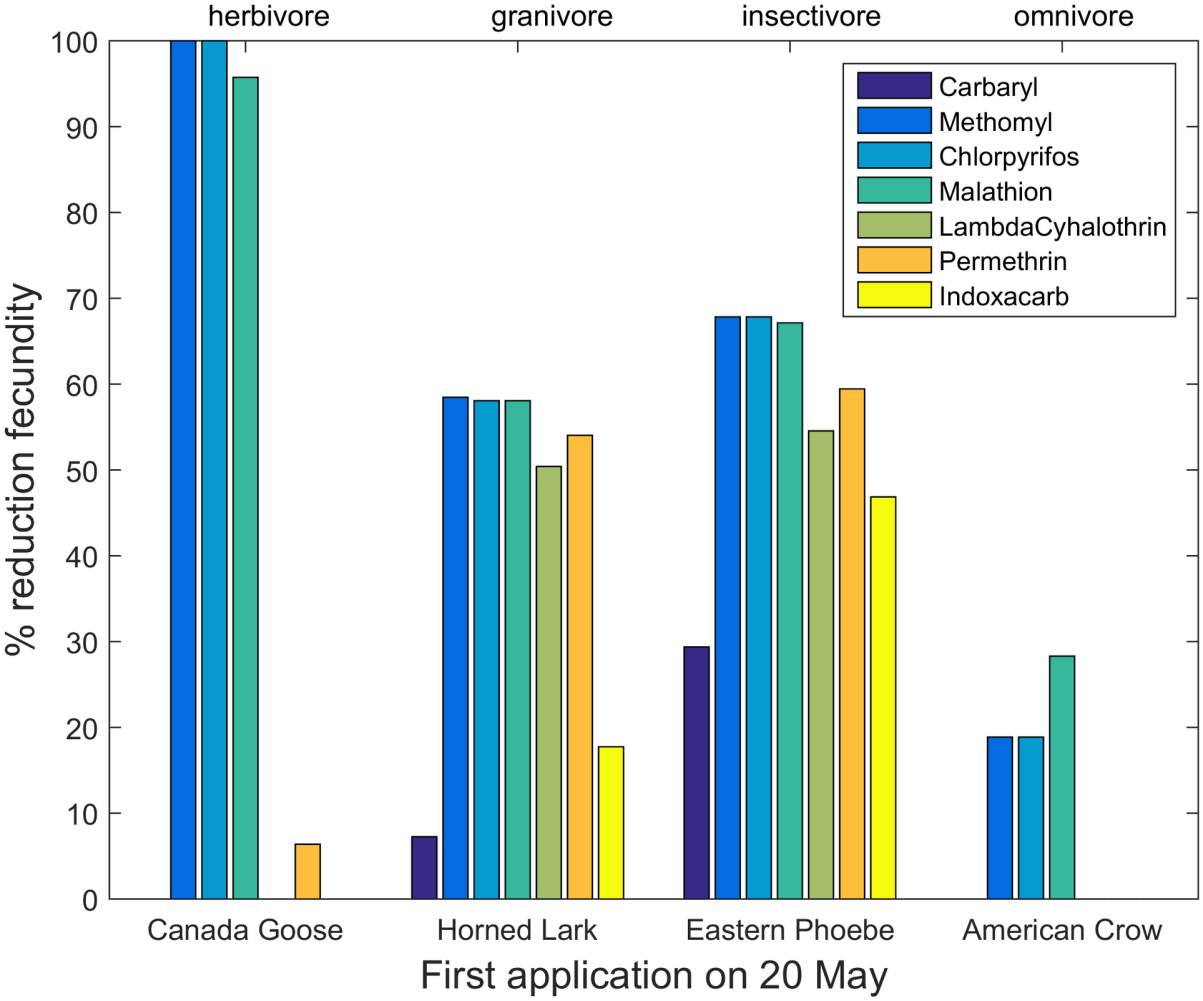
Percent reduction in fecundity for four early-nesting species with first application date occurring on 20 May.

**Fig 4:**
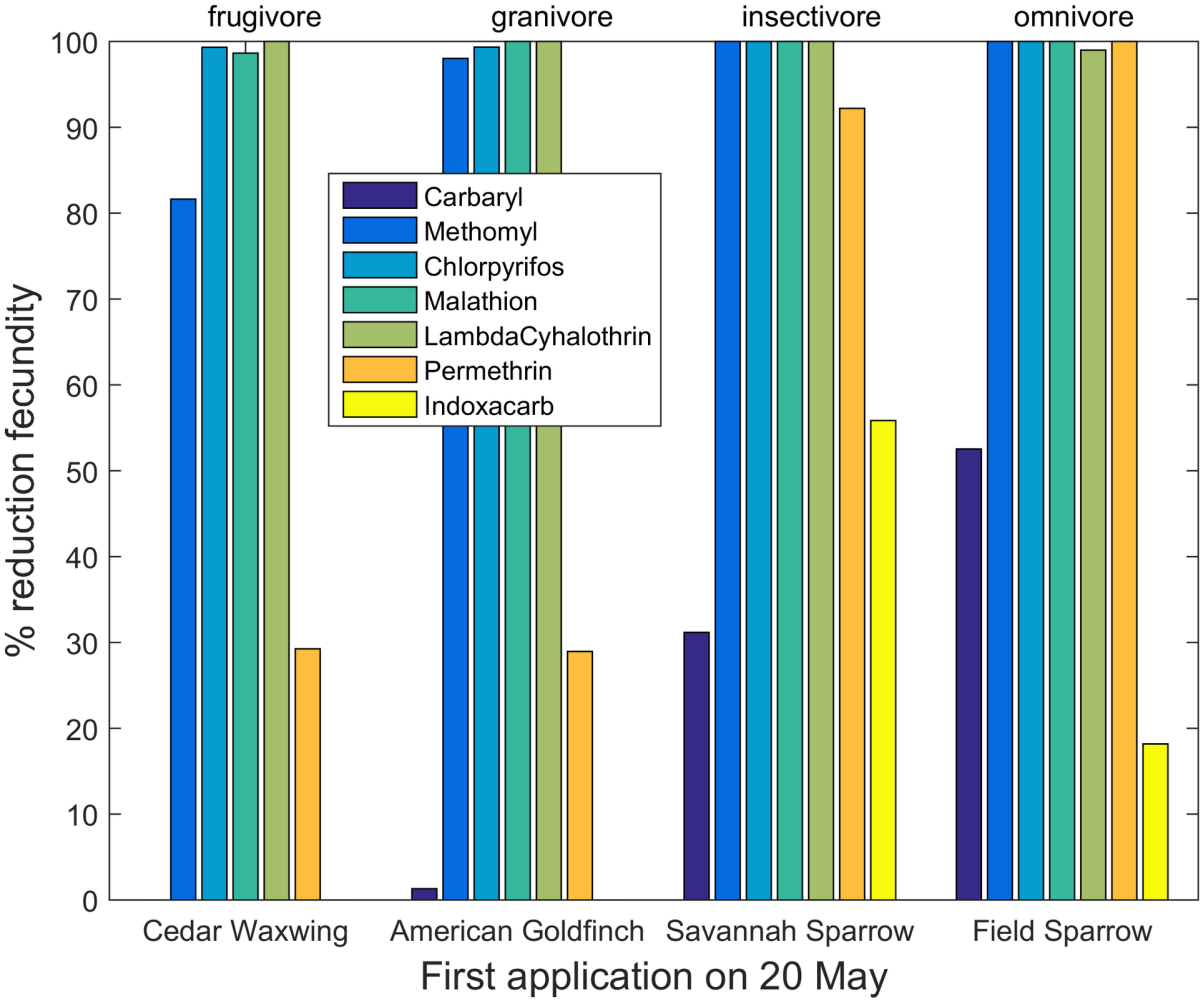
Percent reduction in fecundity for four late-nesting species with first application date occurring on 20 May.

**Fig 5:**
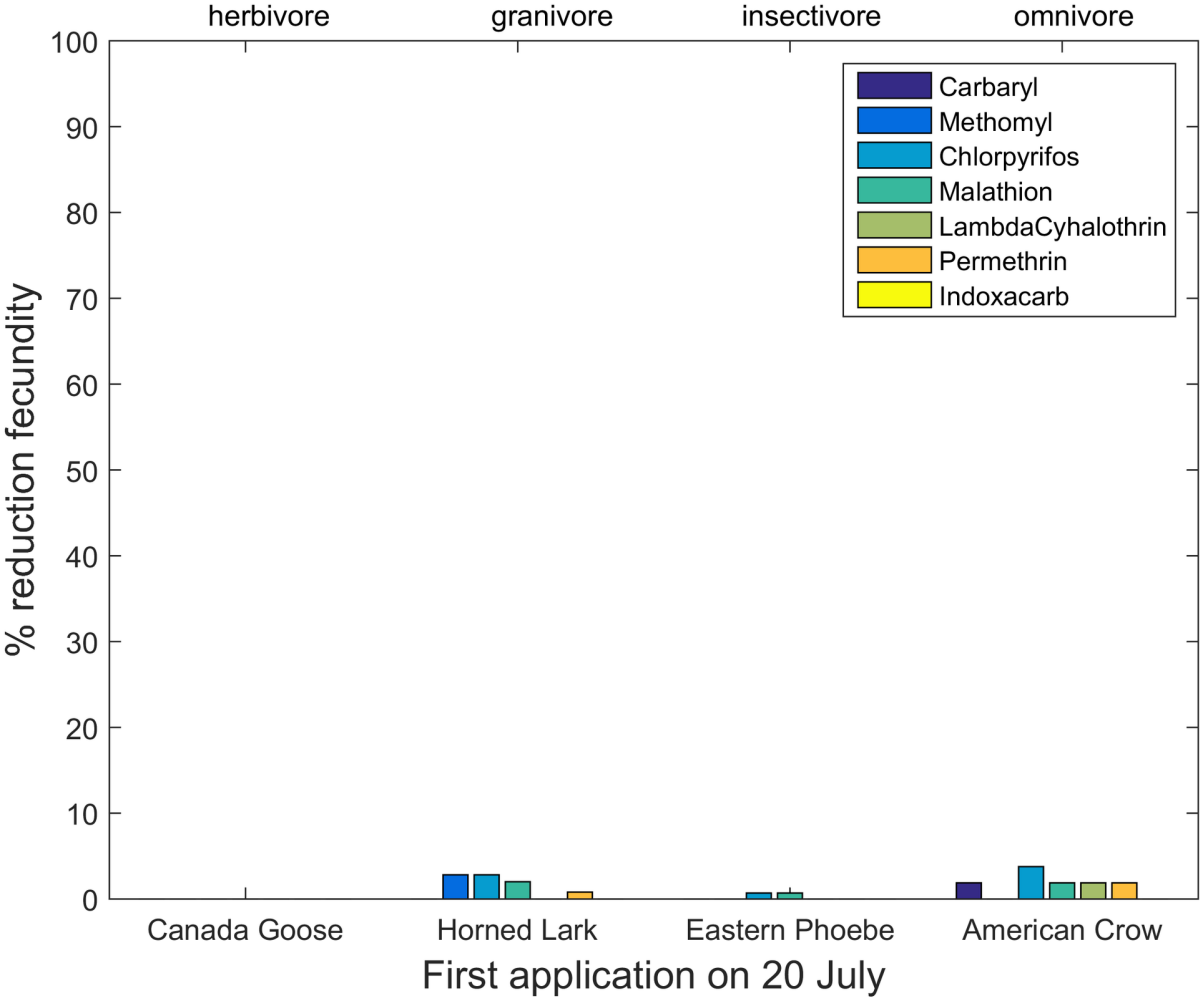
Percent reduction in fecundity for four early-nesting species with first application date occurring on 20 July.

**Fig 6:**
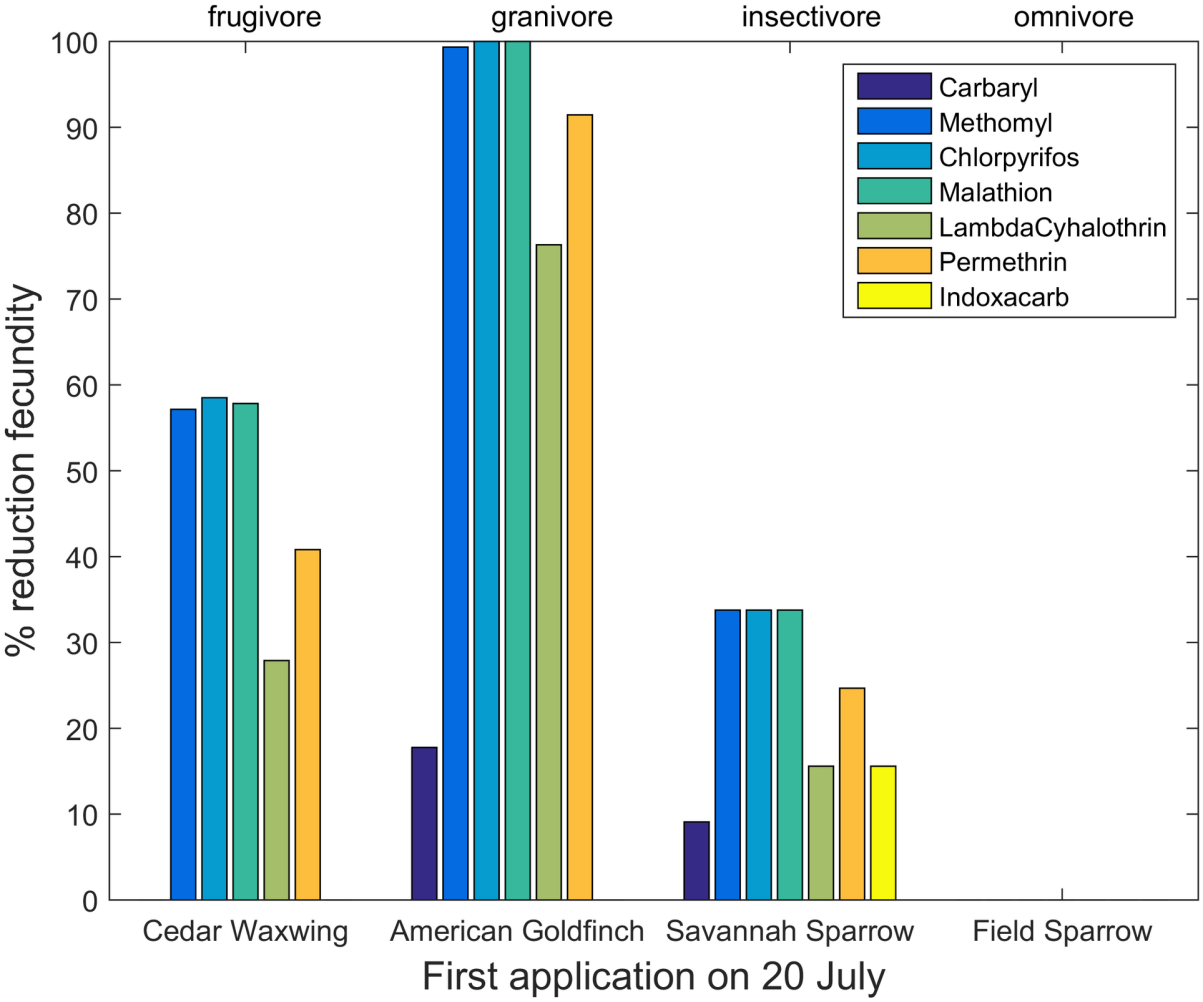
Percent reduction in fecundity for four late-nesting species with first application date occurring on 20 July.

Reductions in fecundity differed among insecticides, species and application dates (Tables H—N, Fig B in S4 Appendix). The largest reductions were observed in the organophosphate insecticides, but all insecticides showed some reductions in reproductive success for certain species. Model results also demonstrated changing patterns of risk as the season progressed, but the relationship between risk and timing differed among species. Fig 3 shows reductions in reproductive success for early nesting species after a first insecticide application on 20 May, whereas Fig 4 shows patterns for late-nesting species exposed to the same application dates. The early-nesting species suffer far lower reductions in reproductive success than do the late-nesting species. However, this pattern changed with the date of first application. Figs 5 and 6 show percent reductions for the same species depicted in Figs 3 and 4, but with the first application occurring on 20 July. In this case, the early-nesting species are able to complete their nesting seasons prior to the first application and achieve almost their full expected reproductive success. However, the late-nesting species still suffer considerable reductions in fecundity. Figures and tables giving reductions in predicted fecundity for all species under all application dates are provided in the Supporting Information (S4 Appendix). As with mortality, model results also suggest differential effects on reproductive success depending on the primary diet of the species modeled (Figs 3–6).

### Sensitivity analysis

**Fig 7:**
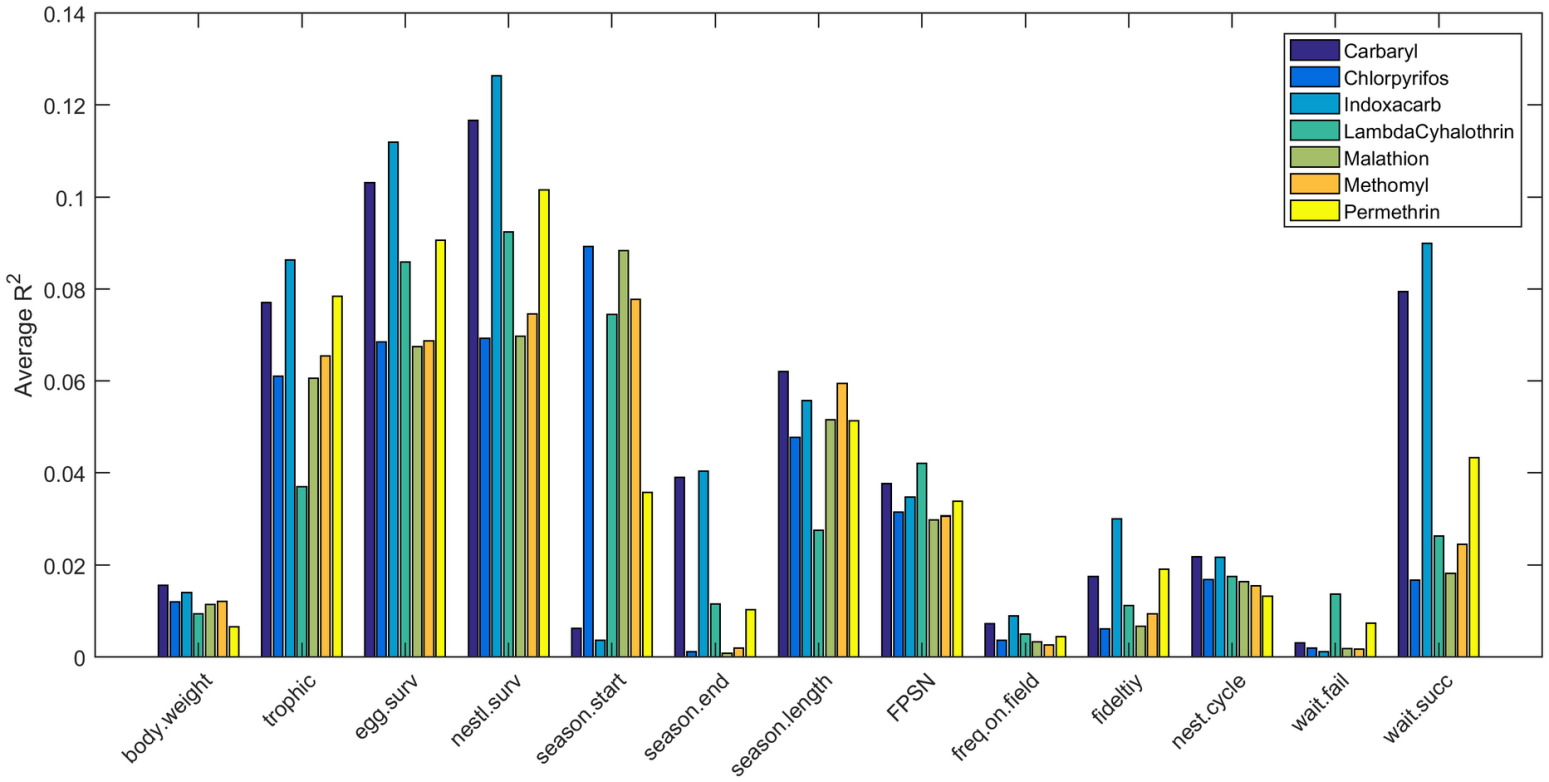
Average R^2^ for linear regressions of fecundity on 13 model parameters averaged across four different first application dates (May 20, June 20, July 20, and August 20).

**Fig 8:**
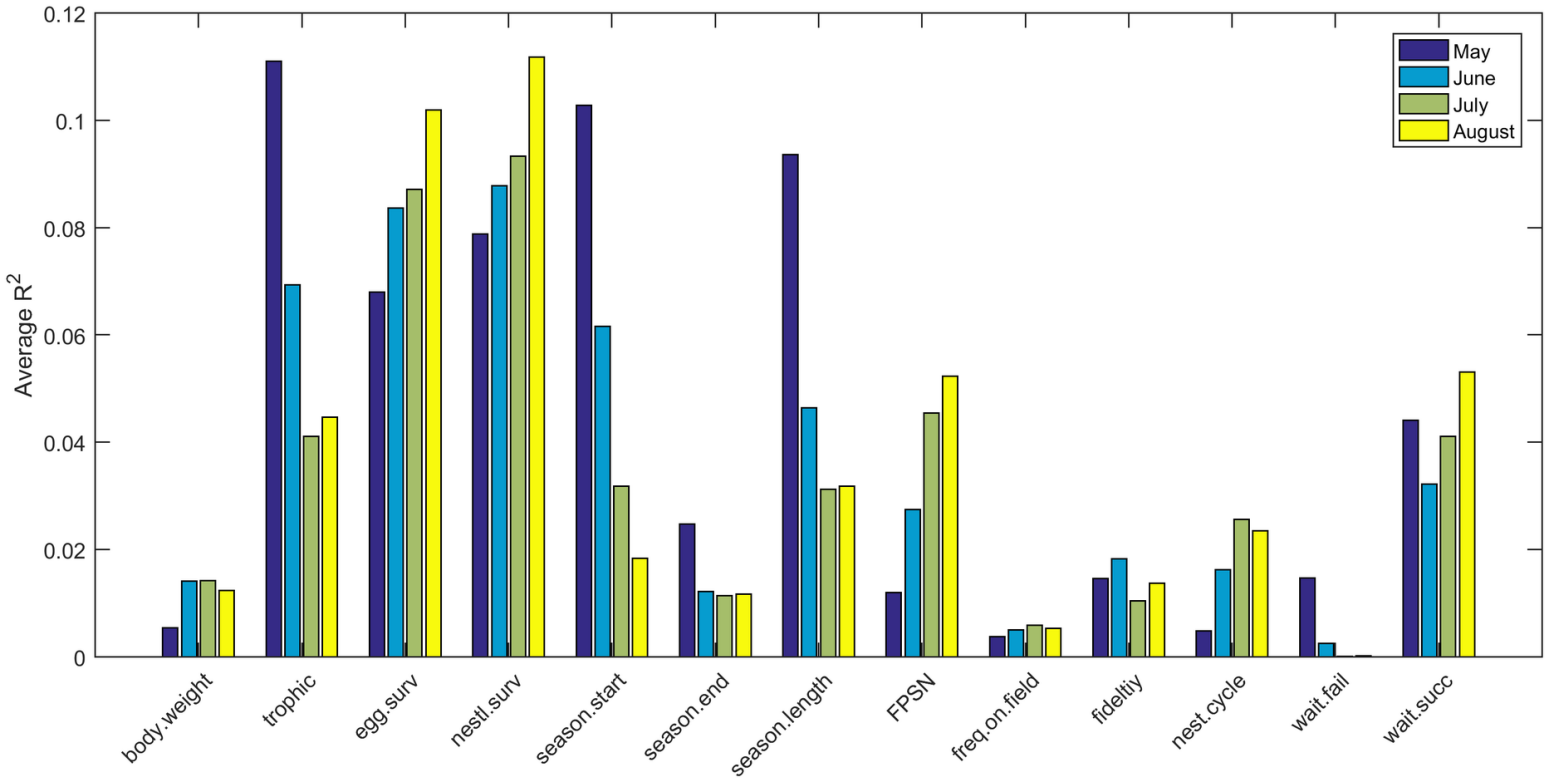
Average R^2^ for linear regressions of fecundity on 13 model parameters averaged across seven pesticides.

Model sensitivity varied widely for different ecological traits. Fig 7 shows the proportion of variance explained (R^2^) by each of 13 model parameters for each of the 7 insecticides (averaged across month of first application). The most important parameters are egg and nestling survival rates, which explained between 6 and 13% of variance in simulated reproductive success. Also important were trophic status, season length, and the number of fledglings produced per successful nest. Two parameters (length of waiting period between a successful nest and the next nest attempt, and the date of the start of the nest season) showed high variability in relative amount of variance explained among chemicals. Similar results were obtained when comparing R^2^ between months of first application (averaged across insecticide, Fig 8). The nest survival parameters again proved to be the most important, explaining between 7 and 11% of the observed variance in reproductive success. Perhaps not surprisingly, the amount of variance explained by the date of the start of the breeding season and the length of the breeding season varied strongly depending on the month of first application. The number of fledglings per successful nest and the length of the waiting period after a successful nest were of intermediate importance when R^2^ was summarized by month (Fig 8).

## Discussion

Model results showed clear differences in predicted risk among insecticides. Further, risk projections also differed among species exposed to the same insecticide. Results from the TIM/MCnest model offer additional insight, compared to RQs, when information about the magnitude of risk or the probability of adverse effects [17,42,43] is needed for higher tier risk assessments. Another important advantage of the model we describe here is that it allows risk assessors to rationally combine both acute and chronic effects into a single unified measure of risk (percent reduction in annual fecundity) that gives appropriate weights to acute versus chronic effects. This unified measure of risk depends on the ecological traits of the species for which risk is being estimated. Ecologists have repeatedly shown that changes in survival versus fecundity can have very different impacts on population growth depending on the life history of a species [44]. However, standard RQ-based methods require USEPA risk assessors to compare RQs to fixed levels of concern, typically 0.5 for acute effects and 1.0 for chronic effects. Thus the Tier 1 approach assigns fixed *a priori* weights to acute versus chronic effects, without taking into account the sensitivity of the species under consideration to changes in survival versus fecundity.

Another advantage of the integrated TIM/MCnest model is that it provides results (fledglings per female per breeding season) that can be used directly as an input for population models to predict population-level response to insecticide exposure in birds. This methodological need has long been identified [45–47], but has been difficult to implement [43], especially given the limitations to standard toxicity data [16,17,42]. The model does so by translating standard toxicity data into endpoints that are relevant to life cycles of birds. The inclusion of a detailed model of avian breeding allows users to explore hypotheses about the mechanisms of effects [48] of insecticide exposure on different components of avian breeding, such as embryo viability, nestling survival, or parental provisioning. These models fit well within the adverse outcome pathway paradigm [7,49] for extrapolating effects from lower levels of biological organization up through their potential population-level consequences.

Sensitivity analyses showed that several ecological traits proved particularly important for predicting vulnerability to insecticide impacts, including nest survival rates, the number of fledglings produced per successful nest, trophic class, and the timing of breeding in relation to insecticide exposure. These parameters are generally known and can be found for many species in standard references such as the Birds of North America [50]. Thus, this model can also be used to help identify important suites of life-history traits that make birds vulnerable to adverse effects of insecticides. However, it should be noted that the particular sensitivity results we found are specific to the application scenario and set of species used in our analyses; different results might be obtained under different application scenarios and/or species sets. TIM/MCnest can also be used for site-specific risk assessments or assessments of listed species to identify those species for which higher-tier risk assessments may be required. Finally, the integrated model should prove useful for managing risk by elucidating the relationship between timing of insecticide use and adverse reproductive effects, potentially allowing labeling restrictions to mitigate risk to sensitive or listed species.

### Model validity

The scientific literature on validation of ecological models is confusing and often contradictory [51,52]. While the idea of challenging model predictions against empirical data is perhaps the most common notion of model validation [51], it is rarely possible and in many cases is not necessary to validate the use of an ecological model [51,52]. However, many different types of activities fall under the general description of model evaluation [51,52]. Augusiak et al. [52] highlight the importance of precisely defining the purpose of a model and the performance criteria against which it will be judged before any real evaluation can take place. Below, we describe some of the many different ways in which TIM/MCnest is evaluated.

For every release of MCnest, the code undergoes an evaluation to verify that the calculations performed by the model are as intended. This is accomplished by examining the progress of individual birds through the various reproductive phases. This progression depends upon the input parameters, which determine the distribution of days over which specific developmental events could occur. Nest failures, due to exceedances of the phase-specific endpoints, are also examined and compared to specific exposure profiles to ensure that modeled pesticide-induced failures occur when intended in the model.

Several algorithms and subroutines in MCnest have been tested against field data. Etterson et al. [24] compared two versions of the parameterized MCnest model to field-based predictions of the number of broods per female produced in Illinois populations of Eastern meadowlark (*Sturnella magna*) and dickcissel (*Spiza americana*). The Markov algorithm behind MCnest has also been adapted for use as a multistate competing risks estimation model and has been applied to the problem of estimation of stressor effects on wild bird populations [53–55], including two cases exploring the effects of environmental contaminants [56,57]. Etterson [4] also showed how the framework could be further adapted to estimate acute mortality of contaminants in field trials when carcass scavenging and imperfect detection make inference difficult. Thus, the algorithms behind TIM/MCnest have been validated in diverse applications under a wide variety of data conditions.

Many of the exposure and effects components of TIM are based on empirical data sets. Model results are evaluated in the context of chemical-specific toxicity and exposure information, as well as available field studies and avian incident reports. For example, for the carbamate carbofuran, TIM predicted a high probability of mortality for several focal species. Concordant avian mortalities were also observed in carbofuran field studies and incident reports [58].

### Model uncertainties

The integrated TIM/MCnest model captures important information about pesticide exposure and effects and integrates these with knowledge of the life-history of different avian species. However, several important uncertainties remain. Below we review some of these uncertainties and indicate how they are treated in the integrated model and, where possible, make suggestions about how their treatment likely influences model predictions.

With regards to exposure, there is substantial variation in insecticide residues on dietary items in treated fields [59,60] resulting from variation in, for example, wind conditions, location of food items in the canopy or on the ground, and amount of vegetative cover. For non-invertebrate food items (leaves and grains), data on mean and distribution of residues are used. For invertebrates, it is assumed that residues on insects represent values present on the 90^th^ percentile of agricultural fields. This assumption may result in overestimation of exposure for insectivores across a population. Behavioral mechanisms, such as regurgitation and avoidance, may also influence exposure. These behaviors are not included in the integrated model and could result in reduced exposure. However, both regurgitation and avoidance could have substantial adverse effects themselves if they result in increased difficulty or effort required to obtain sufficient rations.

In estimating exposures to birds, TIM does not require explicit field dimensions. The model simulates avian mortality that is conditional on exposure. It is assumed that birds move on and off of the treated field to obtain food resources and to nest. The proportion of time spent on the field (where exposure is greatest) is described by “frequency on field” estimates that are based on avian census studies [10,15,19,20] and provided in the Supporting Information (S1 Appendix). As indicated by available avian census studies, use of fields by individuals within the same species varies in time and location. This may be due to differences in field dimensions or characteristics of the adjacent habitats.

A growing body of research suggests that indirect effects (e.g., due to declines in available prey) may be important drivers of population response to large-scale insecticide usage [6,61–64]. Thus, survival and/or reproduction could be adversely affected by pesticide usage even without inducing a direct toxicological response in birds. Parameterization of such effects would require considerable additional information concerning prey capture rates and the relationship between food and energy acquisition and vital rates. Thus, indirect effects are not currently accounted for in TIM/MCnest.

MCnest uses phase-specific thresholds to determine whether a nest attempt is adversely affected by an insecticide during a given developmental phase. If a phase-specific threshold is exceeded, then it is assumed in MCnest that the entire nest fails. In reality, it is possible that only a subset of eggs/nestlings would be lost. However, the current design of avian reproduction studies does not allow proper fitting of dose-response curves to endpoints measured in the avian reproduction test [42,45]. These tests are designed to identify NOAELs using Analysis of Variance in a hypothesis testing framework. Thus, the current design of MCnest is appropriately constrained to the quality and structure of available data on reproductive toxicity. The assumption of complete nest failure (rather than brood reduction) with threshold exceedance appears conservative, but may not be under some conditions. For example, a bird that uses a complete nest attempt to raise a single fledgling (i.e., due to brood reduction) may actually experience lower reproductive success than a bird that loses an entire nest attempt, but subsequently renests and raises a full brood. Analyses and simulations (unpublished) suggest that the assumption of complete loss of nest contents upon threshold exceedance would generally be conservative.

Another important area of model uncertainty arises from our limited knowledge of sensitivity among birds for most insecticides. Under FIFRA, results are generally available for only two species, Northern bobwhite (*Colinus virginianus*, Galliformes) and mallard (*Anas platyrhynchos*, Anseriformes). Acute toxicity (LD_50_) is scaled using body weight and, where available, empirical scaling factors [23]. When acute oral toxicity data are available for additional test species, it may be possible to derive a species sensitivity distribution to evaluate uncertainty associated with the LD50 used by TIM. MCnest currently makes no modification to endpoints from the avian reproduction test to account for interspecific variability. Luttik et al. [65] summarize possible approaches for addressing interspecies variability in effects on avian reproductive endpoints and recommend a method for estimating extrapolation factors proposed by Luttik and Aldenberg [66,67]. These methods may be implemented in a future version of MCnest and users could implement them in the current version by pre-processing the reproduction NOAEL’s following the described methodology.

### Model qualification

Qualification (defining the domain over which a model is considered valid) is an important component to model validation [51]. The version of TIM/MCnest presented here is qualified under a limited set of conditions. Several important qualifications are listed below, but in the interest of brevity, we make little attempt at elaborating beyond the implications for the work presented here. TIM is currently qualified for insecticides and other pesticides distributed via spray application only; this prevented us from considering seed treatments (e.g., neonicotinoids) and granular formulations (e.g., terbufos). TIM can model birds whose diet consists of some combination of seeds, fruit, invertebrates, and foliage; however, it cannot accommodate piscivores, nectarivores, vertebrate predators, or scavengers. TIM cannot model exposure through dermal exposure in aquatic systems, exposure through soil ingestion, or oral uptake through preening or nest-building. In addition, TIM/MCnest is qualified for single a.i. applications at a time, a qualification that prevented us from considering environmental or tank mixtures. MCnest is limited to a subset of avian life histories, specifically terrestrial passerines (or non-passerines with passerine-like nesting ecology) with temporally limited breeding seasons. The list of species in the current MCnest library [28] is generally representative of the life histories for which MCnest is qualified.

### Areas for future research

The results presented above also suggest areas where further research would be useful. For example, model design for both TIM and MCnest is strongly constrained by the structure and design of current toxicity tests. In particular, the endpoints from the avian reproduction test, as NOAELs, limit the nature of inference that can be drawn from them. Modifying the test has both scientific and policy implications that would need to be considered very carefully. Redesigning the avian reproduction test to produce effect-response curves and to enable specification of Effect Concentration values (ECx) would require considerable additional research. For example, having a concentration-response curve for egg-shell thinning, which is clearly and historically related to pesticide effects on avian reproductive success [68], would not necessarily prove informative without associated research on eggshell thickness and probability of egg breakage during incubation. Yet current test designs for avian reproduction do not include incubation, but specify that eggs are removed from females and artificially incubated. Similar issues would arise with other endpoints from the reproduction test, such as the number of 14-day old chicks per hen [42].

Other potential avenues for future research include re-evaluation of assumptions described above, such as improving our understanding of the distribution of insecticide residues on dietary items following different insecticide application methods, better understanding of residue dissipation under varying environmental conditions, and a better understanding of the variation in ecological traits of non-target species using agricultural ecosystems. Finally, a better understanding of how acute and chronic toxicity interacts with other natural stressors such as food limitation, competition, predation, and nest parasitism would improve our ability to translate laboratory studies to wild populations.

## Conclusions

The integrated TIM/MCnest model is a promising tool to evaluate and compare the risk of insecticides to birds using agroecosystems. The integrated model uses data that are generally available, including information required as part of the FIFRA registration process as well as ecological trait data from the avian scientific literature. TIM/MCnest is another example of how mechanistic models can improve our understanding of the risk of environmental contaminants to wildlife by allowing risk assessors to explore specific hypotheses about the mechanism of action or adverse outcome pathways leading to individual impairment and population level demographic challenges. As such, models like TIM/MCnest offer significant improvements to the information provided by risk quotients, which convey little or no data on the magnitude and probability of risk. Nevertheless, important data gaps and uncertainties remain, and suggest areas where further research is needed. An important advantage of TIM/MCnest is that it allows risk assessors to rationally combine both acute and chronic effects into a single unified measure of risk. The relative contribution of acute (lethal) and chronic (reproductive) effects to the unified risk measure depends on the life history and ecological traits of the species rather than *a priori* levels of concern. The integrated TIM/MCnest model, which uses fate and toxicity data, is a promising start towards true population-level risk assessment for birds exposed to insecticides.

## Supporting information

S1 Appendix Tier I analysis using T-Rex. (XLSX)

S2 Appendix Species library for 31 species used in analysis. (DOCX)

S3 Appendix Input data used for TIM/MCnest. (DOCX)

S4 Appendix Full results for all simulations. (DOCX)
