# Supplementary material for: Mechanistic modeling of insecticide risks to breeding birds in North American agroecosystems
Source: PLoS One. 2017 May 3;12(5):e0176998. doi: 10.1371/journal.pone.0176998 (PMC5415183; doi:10.1371/journal.pone.0176998)
Supplement: S2 Appendix — (DOCX) [file pone.0176998.s002.docx]

Current USEPA risk assessments for pesticides generally rely on endpoints that are based on laboratory based toxicity studies focused on groups of individuals. S1 Appendix. Tier I analysis using T-Rex.

**Tier I analysis**

Risk Quotients (RQs) were generated using the T-REX model for the pesticides listed in Table 2, using their associated application parameter values (*i.e.,* rate, number of applications and interval) as well as the most sensitive acute and chronic endpoints available (S1, S2 Tables) and foliar dissipation half-lives (S2 Table). These RQs consider exposures through consumption of contaminated diets that are directly on the field.

When considering the twelve pesticides listed in Table 1, carbaryl, chlorpyrifos, indoxacarb, malathion, and methomyl have acute, dose-based RQs that exceed the level of concern (LOC) of 0.5 for one or more dietary strategies (Table S1). The pesticides that have RQs indicating potential risk of mortality are indoxacarb and acetylcholinesterase inhibitors (organophosphates or carbamates).

Chronic RQs for birds exposed to carbaryl, chlorpyrifos, λ-cyhalothrin, malathion, methomyl or permethrin also exceed the LOC (1.0) (Table S2). The chemicals with RQs indicating potential risk of reproductive effects also include the organophosphates, carbamates and pyrethroids.

Estimated exposures are greatest for insectivores, followed by frugivores and granivores. Therefore, in cases where a chemical’s RQ for insectivores exceeds the LOC, the RQ for frugivores and granivores do not necessary indicate a concern (*e.g.,* malathion). This allows for identification of potential life history characteristics (*i.e.,* diet) of a species that may lead to differences in susceptibility among avian species.

The pesticides with acute and chronic RQs below the LOCs pass the Tier I risk assessment, indicating low concern for risk of mortality or reproductive effects. Pesticides that pass the screen include bifenthrin, chlorantraniliprole, cyfluthrin, esfenvalerate and methoxyfenozide.

Table A. Acute, dose-based RQs for 20 g bird generated using T-REX.

| **Pesticide** | **LD50**  **(mg a.i./kg-bw)** | **Test species***** | **Mineau scaling factor** | **Herbivore (short grass) RQ** | **Insectivore RQ** | **Granivore RQ** | **Frugivore RQ** |
| --- | --- | --- | --- | --- | --- | --- | --- |
| bifenthrin | 1800 | quail | 1.15* | 0.021 | 0.0083 | 0.00029 | 0.0013 |
| carbaryl | 2290 | J. quail | 1.5518 | **0.86**** | 0.34 | 0.012 | 0.054 |
| chlorantraniliprole | >2250 | quail | 1.15* | 0.029 | <0.011 | <0.00040 | <0.0018 |
| chlorpyrifos | 8.41 | pheasant | 1.1573 | **76**** | **30**** | **1.1**** | **4.7**** |
| cyfluthrin | >2000 | quail | 1.15* | 0.027 | 0.011 | 0.00038 | 0.0017 |
| esfenvalerate | 381 | quail | 1.15* | 0.18 | 0.072 | 0.0025 | 0.011 |
| indoxacarb | 98 | quail | 1.15* | **0.88**** | 0.34 | 0.012 | 0.055 |
| λ-cyhalothrin | 3950 | mallard | 1.15* | 0.015 | 0.0057 | 0.00020 | 0.00092 |
| malathion | 167 | pheasant | 1.15* | **4.6**** | **1.8**** | 0.064 | 0.29 |
| methomyl | 15 | pheasant | 1.0778 | **46**** | **18**** | 0.64 | **2.9**** |
| methoxyfenozide | >2250 | quail | 1.15* | 0.15 | <0.057 | <0.0020 | <0.0091 |
| permethrin | >9869 | mallard | 1.15* | 0.056 | <0.022 | <0.00078 | <0.0035 |

*Default

**Exceeds acute (0.5) level of concern

***quail = northern bobwhite quail (*Colinus virginianus*); J. quail = Japanese quail (*Coturnix japonica*); mallard = mallard duck (*Anas* ***platyrhynchos*); pheasant = ring-necked pheasant (*Phasianus colchicus*)**

Table B. Chronic, dietary-based RQs generated using T-REX.

| **Pesticide** | **Foliar dissipation**  **half-life (d)** | **NOEC**  **(mg a.i./kg)** | Herbivore  (short grass ) RQ | Insectivore  RQ | **Granivore and**  **Frugivore RQ** |
| --- | --- | --- | --- | --- | --- |
| bifenthrin | 8.3 | 75 | 0.32 | 0.13 | 0.020 |
| carbaryl | 3.71 | 300 | **1.7******** | 0.041 | 0.0065 |
| chlorantraniliprole | 35* | 120 | 0.34 | 0.0031 | 0.00049 |
| chlorpyrifos | 4.0 | 25 | **12******** | **4.5******** | 0.72 |
| cyfluthrin | 35 | 250 | 0.99 | 0.39 | 0.062 |
| esfenvalerate | 12.5 | 608 | 0.073 | 0.028 | 0.0045 |
| indoxacarb | 22.46 | 144 | 0.38 | 0.15 | 0.024 |
| λ-cyhalothrin | 35** | 5 | **5.3******** | **2.1******** | 0.33 |
| malathion | 6.1 | 110 | **3.2******** | 0.039 | 0.0062 |
| methomyl | 2.5 | 150 | **2.9******** | **1.1******** | 0.025 |
| methoxyfenozide | 35** | 819 | 0.25 | 0.014 | 0.0022 |
| permethrin | 15.4 | 125 | **2.0******** | 0.0099 | 0.0016 |

*Default assumption

**Exceeds chronic level of concern (1.0)
