# Supplementary material for: Mechanistic modeling of insecticide risks to breeding birds in North American agroecosystems
Source: PLoS One. 2017 May 3;12(5):e0176998. doi: 10.1371/journal.pone.0176998 (PMC5415183; doi:10.1371/journal.pone.0176998)
Supplement: S3 Appendix — (DOCX) [file pone.0176998.s003.docx]

S3 Appendix. Input data for TIM/MCnest

Table A. TIM parameters fixed across all pesticides.

| Parameter | Value |
| --- | --- |
| Model dietary exposure | yes |
| Model exposure through drinking from puddles | yes |
| Model exposure through drinking from dew | yes |
| Model exposure through dermal contact with foliage | yes |
| Model exposure through dermal contact with spray | yes |
| Model exposure off-field through spray drift | yes |
| Time of first application | 8:00 AM |
| droplet spectrum | very fine to fine |
| Spray duration (min) | 1.5 |
| Crop height (m) | 0.152 |
| Plant(crop) mass (kg/ha) | 39 |
| crop type | field |
| Fraction of edge habitat receiving spray drift | 1 |
| Length of in field buffer (feet) | 0 |
| fraction of organic carbon in soil | 0.015 |
| soil bulk density (kg/L) | 1.5 |
| Morning feeding start times: min and max | 4:00 am, 5:00 am |
| Morning feeding end times: min and max | 6:00 am, 10:00 am |
| afternoon feeding start times: min and max | 4:00 pm, 7:00 pm |
| afternoon feeding end times: min and max | 8:00 pm, 9:00 pm |
| Proportion of daily feeding taking place in morning: min and max | 0.4, 0.6 |
| Gorging factor | normal feeding |
| Contaminated fraction of food | 1 |
| Dislodgable foliar residue adjustment factor | 0.62 |
| Dermal adsorption fraction | 1 |
| avian acute inhalation LD50 (mg a.i.kg-bw) | unavailable |
| Chemical specific avian dermal LD50 | unavailable |
| Food matrix adjustment factor | 1 |
| ratio of juvenile to adult toxicity | 1 |

Table B. TIM parameters that varied among pesticides

| Pesticide | Carbaryl | Chlorpyrifos | Indoxacarb | λ-Cyhalothrin | Malathion | Methomyl | Permethrin |
| --- | --- | --- | --- | --- | --- | --- | --- |
| Model exposure through vapor inhalation | yes | yes | yes | yes | yes | yes | no |
| Model exposure through spray inhalation | yes | yes | yes | yes | yes | yes | no |
| Number of applications | 4 | 3 | 4 | 4 | 2 | 5 | 5 |
| Application method (spray) | Aerial | Aerial | Ground | Aerial | Aerial | Aerial | Aerial |
| Spray height | 3 m | 3 m | 0.5 m | 3 m | 3 m | 3 m | 3 m |
| Rate of application #1 (lb a.i.A) | 2 | 1 | 0.065 | 0.03 | 1 | 0.45 | 0.24 |
| Interval between app1 and 2 (days) | 14 | 10 | 7 | 7 | 7 | 7 | 7 |
| Rate of application #2 (lb a.i.A) | 2 | 1 | 0.065 | 0.03 | 1 | 0.45 | 0.24 |
| Interval between app2 and 3 (days) | 14 | 10 | 7 | 7 | 0 | 7 | 7 |
| Rate of application #3 (lb a.i.A) | 2 | 1 | 0.065 | 0.03 | 0 | 0.45 | 0.24 |
| Interval between app3 and 4 (days) | 14 | 0 | 7 | 7 | 0 | 7 | 7 |
| Rate of application #4 (lb a.i.A) | 2 | 0 | 0.065 | 0.03 | 0 | 0.45 | 0.24 |
| Interval between app 4 and 5 (days) | 0 | 0 | 0 | 0 | 0 | 7 | 7 |
| Rate of application #5 (lb a.i.A) | 0 | 0 | 0 | 0 | 0 | 0.45 | 0.24 |
| Food item half-lives (days) | 3.71 | 4 | 22.46 | 35 | 6.1 | 2.5 | 15.4 |
| Pesticide half-life (days) in puddle | 12 | 170.6 | 156 | 76.2 | 15.9 | 59.4 | 101 |
| K_oc_ (Lkg-oc) | 2.575 | 6040 | 1700 | 333200 | 217 | 46 | 29420 |
| K_ow_ | 229 | 50118 | 44668 | 1.00E+07 | 799 | 20.4 | 1258925 |
| Henry's law constant (atm*m^3^/mol) | 1.28E-08 | 6.20E-06 | 6.00E-10 | 1.90E-07 | 1.20E-07 | 2.10E-11 | 1.40E-06 |
| solubility in water (mg a.i./L) | 32 | 1.40 | 0.2 | 0.005 | 145 | 5.50E+04 | 0.0055 |
| avian acute oral LD50 (mg a.i.kg/bw) | 20,000 | 29.2 | 98 | 50000 | 359 | 24.2 | 112,740 |
| Body weight of tested animals | 968.5 | 178 | 178 | 1049 | 195 | 178 | 1580 |
| slope of avian oral LD50 | 10 | 4.5 | 6.24 | 10 | 9.42 | 5.33 | 10 |
| Mineau scaling factor | 1.5518 | 1.1573 | 1.15 | 1.15 | 1.15 | 1.0778 | 1.15 |
| Rat inhalation LD50 (mg a.i.kg/bw) | 237 | 190 | 539 | 229 | 310 | 13 | 340.5 |
| rat acute oral LD50 (mg a.i.kg/bw) | 310 | 137 | 5000 | 56 | 5400 | 7.14 | 340.5 |
| Hourly fraction of pesticide retained | 0.994 | 0.995 | 0.992 | 0.998 | 0.986 | 0.996 | 0.912 |

Table C. TIM parameters that varied among species

| Parameter |
| --- |
| Passerine vs. Non-passerine |
| Altricial vs. precocial |
| Body Weight |
| Body weight (g): mean, SD, min, max |
| feeding category: ( insectivore, herbivore, granivore, omnivore) |
| Fraction of each food item in diet (insects, seeds, fruit, grass, broadleaf) |
| For juveniles: fraction of each food item in diet (insects, seeds, fruit, grass, broadleaf) |
| Resident status (field vs. edge) |
| Respiratory physiology adjustment factor |
| Frequency on field |
| Fidelity factor |
| Passerine |

Table D. Avian reproduction test data for seven modeled pesticides

| Pesticide | Carbaryl | Chlorpyrifos | Indoxacarb | λ-Cyhalothrin | Malathion | Methomyl | Permethrin |
| --- | --- | --- | --- | --- | --- | --- | --- |
| Reproduction Test species: | Bobwhite | Bobwhite | Bobwhite | Mallard | Bobwhite | Bobwhite | Bobwhite |
| Dose levels: | 3 | 3 | 4 | 2 | 3 | 3 | 3 |
| Measured dietary conc. (mg/kg diet): | [280, 924, 2930] | [9.8, 39.2, 123.7] | [28.8, 86.4, 144, 720] | [4.62, 50.8] | [112, 358, 1260] | [64, 153, 459] | [23, 115, 472] |
| Average food consumption (g/bird/day): | [21.72, 22.2, 22.3] | [17, 18, 17] | [20.7, 20.5, 20.1, 21.7] | [225.8, 218.3] | [24, 25, 23] | [27.15, 27.1, 27.25] | [21.6, 23.4, 25.4] |
| Average initial female body weight (g): | [206, 208, 215] | [183, 183, 184] | [204, 205, 207, 205] | [962, 962] | [195, 194, 190] | [191, 192, 197] | [187, 191, 188] |
| Average initial male body weight (g): | [210, 207, 202] | [194, 189, 190] | [213, 207, 208, 211] | [1087, 1075] | [193, 192, 193] | [192, 192, 194] | [194, 190, 191] |
| Average final female body weight (g): | [237, 239, 240] | [212, 203, 197] | [205, 206, 209, 203] | [1135, 1113] | [250, 228, 202] | [219, 219, 219] | [229, 237, 234] |
| Average final male body weight (g): | [219, 223, 213] | [197, 194, 190] | [213, 206, 208, 209] | [1249, 1251] | [221, 219, 206] | [207, 208, 204] | [204, 204, 207] |
| NOAELs |  |  |  |  |  |  |  |
| ^1^Number of eggs laid: | 3 | 2 | 4 | 1 | 2 | 2 | 3 |
| ^1^%Viable eggs of eggs set: | 3 | 3 | 4 | 2 | 2 | 3 | 3 |
| ^1^%Live 3-wk embryos of ^1^viable eggs: | 3 | 3 | 4 | 2 | 3 | 3 | 3 |
| ^1^%hatchlings of live 3-wk embryos: | 3 | 3 | 4 | 2 | 3 | 3 | 3 |
| ^1^%14-d chicks of ^1^hatchlings: | 3 | 3 | 4 | 2 | 3 | 3 | 3 |
| ^1^shell thickness: | 3 | 3 | 4 | 2 | 2 | 3 | 3 |
| ^1^prelaying female weight: | 3 | 3 | 3 | 2 | 3 | 3 | 3 |
| ^1^prelaying male weight: | 3 | 3 | 3 | 2 | 3 | 3 | 3 |

^1^Numbers in these cells refer to testing levels and NOAECs for each endpoint. For example, the NOAEC for number of eggs laid for Carbaryl occurred at 2,930 mg ai/kg food. Dietary doses (mg/kg body weight) are calculated from these food-concentration-based estimates, consumption rates, and body weights.

Table E. Avian LC50 and LD50 test data for seven pesticides modeled using TIM/MCnest

| Pesticide | Carbaryl | Chlorpyrifos | Indoxacarb | λ-Cyhalothrin | Malathion | Methomyl | Permethrin |
| --- | --- | --- | --- | --- | --- | --- | --- |
| Mineau scaling factor: | 1.5518 | 1.1573 | 1.15 | 1.15 | 1.15 | 1.0778 | 1.15 |
| LD50 (mg/kg bwt): | 2,000 | 29.2 | 98 | 5000 | 359 | 24.2 | 11274 |
| LD50 Body Weight (g) | 968.5 | 178 | 178 | 1049 | 195 | 178 | 1580 |
| LD50 Slope | 10 | 4.5 | 6.24 | 10 | 9.42 | 5.33 | 10 |
| LD50 Test species: | Mallard | Bobwhite | Bobwhite | Mallard | Bobwhite | Bobwhite | Mallard |
| LC50: | >5000 | 203 | 808 | 2354 | 3497 | 3714 | >10,000 |
| Fraction of LC50 (i.e., 0.1 or 0.5): | 0.5 | 0.5 | 0.5 | 0.5 | 0.5 | 0.5 | 0.5 |
| Mean body weight (g): | 22.5 | 96.2 | 22.7 | 17.4 | 22.5 | 219.2 | 22.5 |
| Mean food ingestion rate (g/d): | 7.7 | 14.2 | 7.8 | 14.96 | 7.7 | 50.4 | 7.7 |
| LC50 Test Species: | Bobwhite | Mallard | Bobwhite | Bobwhite | Bobwhite | Mallard | Bobwhite |
