# Supplementary material for: Mechanistic modeling of insecticide risks to breeding birds in North American agroecosystems
Source: PLoS One. 2017 May 3;12(5):e0176998. doi: 10.1371/journal.pone.0176998 (PMC5415183; doi:10.1371/journal.pone.0176998)
Supplement: S4 Appendix — (DOCX) [file pone.0176998.s004.docx]

S4 Appendix. Results

Table A. Predicted mortality for 31 species associated with exposure to Carbaryl by date of first pesticide application.

| Species | May | June | July | August |
| --- | --- | --- | --- | --- |
| Canada Goose | 0 | 0 | 0 | 0 |
| Northern Bobwhite | 0 | 0 | 0 | 0 |
| Killdeer | 0 | 0 | 0 | 0 |
| Mourning Dove | 0 | 0 | 0 | 0 |
| Northern Flicker | 0 | 0 | 0 | 0 |
| Eastern Phoebe | 0.0001 | 0 | 0.0001 | 0 |
| Eastern Kingbird | 0 | 0 | 0 | 0 |
| Blue Jay | 0 | 0 | 0 | 0 |
| American Crow | 0 | 0 | 0 | 0 |
| Horned Lark | 0 | 0 | 0 | 0 |
| Barn Swallow | 0.0007 | 0.0001 | 0.0007 | 0.0002 |
| Black-capped Chickadee | 0.0002 | 0.0003 | 0 | 0 |
| House Wren | 0.0023 | 0.0032 | 0.0017 | 0 |
| Eastern Bluebird | 0.0001 | 0 | 0 | 0 |
| American Robin | 0 | 0 | 0 | 0 |
| Cedar Waxwing | 0 | 0 | 0 | 0 |
| Common Yellowthroat | 0.0054 | 0.0057 | 0.0016 | 0 |
| Yellow Warbler | 0.0546 | 0.0199 | 0 | 0 |
| Chipping Sparrow | 0.0001 | 0.0001 | 0.0001 | 0 |
| Field Sparrow | 0.0008 | 0.0008 | 0 | 0 |
| Vesper Sparrow | 0 | 0 | 0 | 0 |
| Savannah Sparrow | 0.0007 | 0.0007 | 0 | 0 |
| Grasshopper Sparrow | 0.0001 | 0.0001 | 0 | 0 |
| Northern Cardinal | 0 | 0 | 0 | 0 |
| Dickcissel | 0 | 0 | 0 | 0 |
| Red-winged Blackbird | 0 | 0 | 0 | 0 |
| Eastern Meadowlark | 0 | 0 | 0 | 0 |
| Western Meadowlark | 0 | 0 | 0 | 0 |
| Common Grackle | 0 | 0 | 0 | 0 |
| American Goldfinch | 0 | 0 | 0 | 0 |
| House Sparrow | 0 | 0 | 0 | 0 |

Table B. Predicted mortality for 31 species associated with exposure to Chlorpyrifos by date of first pesticide application.

| Species | May | June | July | August |
| --- | --- | --- | --- | --- |
| Canada Goose | 0.7283 | 0.7263 | 0.3614 | 0 |
| Northern Bobwhite | 0.7968 | 0.7964 | 0.8023 | 0.8018 |
| Killdeer | 0.999 | 0.9989 | 0.9989 | 0.9987 |
| Mourning Dove | 0.6171 | 0.6334 | 0.6219 | 0.6171 |
| Northern Flicker | 0.8571 | 0.8505 | 0.7621 | 0 |
| Eastern Phoebe | 1 | 1 | 0.999 | 0 |
| Eastern Kingbird | 0.9994 | 0.9994 | 0.9975 | 0 |
| Blue Jay | 0.9071 | 0.6143 | 0 | 0 |
| American Crow | 0.0536 | 0.0047 | 0 | 0 |
| Horned Lark | 1 | 1 | 0.9956 | 0 |
| Barn Swallow | 1 | 1 | 1 | 1 |
| Black-capped Chickadee | 1 | 1 | 1 | 0 |
| House Wren | 1 | 1 | 1 | 0 |
| Eastern Bluebird | 1 | 1 | 1 | 0.994 |
| American Robin | 0.9887 | 0.9884 | 0.9907 | 0.2383 |
| Cedar Waxwing | 0.9901 | 0.9903 | 0.9903 | 0.9905 |
| Common Yellowthroat | 1 | 1 | 0.9999 | 0 |
| Yellow Warbler | 1 | 1 | 0 | 0 |
| Chipping Sparrow | 0.9995 | 0.9996 | 0.9984 | 0 |
| Field Sparrow | 1 | 1 | 0.9916 | 0 |
| Vesper Sparrow | 0.9999 | 0.9999 | 0.9999 | 0 |
| Savannah Sparrow | 1 | 1 | 1 | 0 |
| Grasshopper Sparrow | 1 | 1 | 0.9999 | 0 |
| Northern Cardinal | 0.9909 | 0.9911 | 0.9896 | 0.8834 |
| Dickcissel | 1 | 1 | 1 | 0 |
| Red-winged Blackbird | 0.9985 | 0.9986 | 0.998 | 0 |
| Eastern Meadowlark | 1 | 1 | 1 | 0 |
| Western Meadowlark | 0.9978 | 0.9977 | 0.998 | 0 |
| Common Grackle | 0.8682 | 0.8574 | 0 | 0 |
| American Goldfinch | 0.9926 | 0.9918 | 0.9925 | 0.9932 |
| House Sparrow | 0.8991 | 0.8962 | 0.899 | 0.8356 |

Table C. Predicted mortality for 31 species associated with exposure to Indoxacarb by date of first pesticide application.

| Species | May | June | July | August |
| --- | --- | --- | --- | --- |
| Canada Goose | 0 | 0 | 0 | 0 |
| Northern Bobwhite | 0 | 0 | 0 | 0 |
| Killdeer | 0.0006 | 0.0007 | 0.0007 | 0.0003 |
| Mourning Dove | 0 | 0 | 0 | 0 |
| Northern Flicker | 0 | 0 | 0 | 0 |
| Eastern Phoebe | 0.1164 | 0.1165 | 0.0001 | 0 |
| Eastern Kingbird | 0.0141 | 0.0161 | 0.0013 | 0 |
| Blue Jay | 0 | 0 | 0 | 0 |
| American Crow | 0 | 0 | 0 | 0 |
| Horned Lark | 0.0028 | 0.0027 | 0 | 0 |
| Barn Swallow | 0.1849 | 0.1894 | 0.1807 | 0.1806 |
| Black-capped Chickadee | 0.0025 | 0.0017 | 0 | 0 |
| House Wren | 0.0616 | 0.0611 | 0.0583 | 0 |
| Eastern Bluebird | 0.1089 | 0.1057 | 0.1027 | 0 |
| American Robin | 0.0013 | 0.0011 | 0.0009 | 0 |
| Cedar Waxwing | 0 | 0 | 0 | 0 |
| Common Yellowthroat | 0.0655 | 0.0645 | 0.0156 | 0 |
| Yellow Warbler | 0.2984 | 0.1213 | 0 | 0 |
| Chipping Sparrow | 0.0015 | 0.0014 | 0.0001 | 0 |
| Field Sparrow | 0.0096 | 0.011 | 0 | 0 |
| Vesper Sparrow | 0.006 | 0.0059 | 0.0037 | 0 |
| Savannah Sparrow | 0.1186 | 0.1171 | 0.1026 | 0 |
| Grasshopper Sparrow | 0.0108 | 0.0102 | 0.0023 | 0 |
| Northern Cardinal | 0.0002 | 0.0001 | 0.0002 | 0 |
| Dickcissel | 0.0088 | 0.0075 | 0.0067 | 0 |
| Red-winged Blackbird | 0.0019 | 0.0031 | 0.0004 | 0 |
| Eastern Meadowlark | 0.027 | 0.0304 | 0.0033 | 0 |
| Western Meadowlark | 0.001 | 0.0015 | 0.0012 | 0 |
| Common Grackle | 0 | 0 | 0 | 0 |
| American Goldfinch | 0 | 0.0002 | 0.0002 | 0.0003 |
| House Sparrow | 0 | 0 | 0 | 0 |

Table D. Predicted mortality for 31 species associated with exposure to λ-Cyhalothrin by date of first pesticide application.

| Species | May | June | July | August |
| --- | --- | --- | --- | --- |
| Canada Goose | 0 | 0 | 0 | 0 |
| Northern Bobwhite | 0 | 0 | 0 | 0 |
| Killdeer | 0 | 0 | 0 | 0 |
| Mourning Dove | 0 | 0 | 0 | 0 |
| Northern Flicker | 0 | 0 | 0 | 0 |
| Eastern Phoebe | 0 | 0 | 0 | 0 |
| Eastern Kingbird | 0 | 0 | 0 | 0 |
| Blue Jay | 0 | 0 | 0 | 0 |
| American Crow | 0 | 0 | 0 | 0 |
| Horned Lark | 0 | 0 | 0 | 0 |
| Barn Swallow | 0 | 0 | 0 | 0 |
| Black-capped Chickadee | 0 | 0 | 0 | 0 |
| House Wren | 0 | 0 | 0 | 0 |
| Eastern Bluebird | 0 | 0 | 0 | 0 |
| American Robin | 0 | 0 | 0 | 0 |
| Cedar Waxwing | 0 | 0 | 0 | 0 |
| Common Yellowthroat | 0 | 0 | 0 | 0 |
| Yellow Warbler | 0 | 0 | 0 | 0 |
| Chipping Sparrow | 0 | 0 | 0 | 0 |
| Field Sparrow | 0 | 0 | 0 | 0 |
| Vesper Sparrow | 0 | 0 | 0 | 0 |
| Savannah Sparrow | 0 | 0 | 0 | 0 |
| Grasshopper Sparrow | 0 | 0 | 0 | 0 |
| Northern Cardinal | 0 | 0 | 0 | 0 |
| Dickcissel | 0 | 0 | 0 | 0 |
| Red-winged Blackbird | 0 | 0 | 0 | 0 |
| Eastern Meadowlark | 0 | 0 | 0 | 0 |
| Western Meadowlark | 0 | 0 | 0 | 0 |
| Common Grackle | 0 | 0 | 0 | 0 |
| American Goldfinch | 0 | 0 | 0 | 0 |
| House Sparrow | 0 | 0 | 0 | 0 |

Table E. Predicted mortality for 31 species associated with exposure to Malathion by date of first pesticide application.

| Species | May | June | July | August |
| --- | --- | --- | --- | --- |
| Canada Goose | 0.0526 | 0.0517 | 0.037 | 0 |
| Northern Bobwhite | 0.7351 | 0.7324 | 0.7365 | 0.7321 |
| Killdeer | 1 | 1 | 1 | 1 |
| Mourning Dove | 0.4818 | 0.4791 | 0.4753 | 0.4789 |
| Northern Flicker | 0.434 | 0.4442 | 0.4432 | 0 |
| Eastern Phoebe | 0.9999 | 0.9999 | 0.9998 | 0 |
| Eastern Kingbird | 0.9877 | 0.9891 | 0.9876 | 0 |
| Blue Jay | 0.787 | 0.6647 | 0 | 0 |
| American Crow | 0.0004 | 0 | 0 | 0 |
| Horned Lark | 1 | 1 | 1 | 0 |
| Barn Swallow | 1 | 1 | 1 | 1 |
| Black-capped Chickadee | 1 | 1 | 1 | 0 |
| House Wren | 1 | 1 | 1 | 0 |
| Eastern Bluebird | 1 | 1 | 1 | 0.9873 |
| American Robin | 0.8091 | 0.8151 | 0.8199 | 0.0063 |
| Cedar Waxwing | 0.9879 | 0.9878 | 0.9858 | 0.986 |
| Common Yellowthroat | 1 | 1 | 1 | 0 |
| Yellow Warbler | 1 | 1 | 0 | 0 |
| Chipping Sparrow | 1 | 1 | 1 | 0 |
| Field Sparrow | 1 | 1 | 0.9987 | 0 |
| Vesper Sparrow | 1 | 1 | 1 | 0 |
| Savannah Sparrow | 1 | 1 | 1 | 0 |
| Grasshopper Sparrow | 1 | 1 | 1 | 0 |
| Northern Cardinal | 0.9754 | 0.9764 | 0.9739 | 0.7211 |
| Dickcissel | 1 | 1 | 1 | 0 |
| Red-winged Blackbird | 0.995 | 0.9953 | 0.9948 | 0 |
| Eastern Meadowlark | 1 | 1 | 1 | 0 |
| Western Meadowlark | 1 | 1 | 1 | 0 |
| Common Grackle | 0.7344 | 0.7365 | 0 | 0 |
| American Goldfinch | 0.9986 | 0.9986 | 0.9989 | 0.9985 |
| House Sparrow | 0.8598 | 0.8612 | 0.8653 | 0.86 |

Table F. Predicted mortality for 31 species associated with exposure to Methomyl by date of first pesticide application.

| Species | May | June | July | August |
| --- | --- | --- | --- | --- |
| Canada Goose | 0.5119 | 0.5106 | 0.1997 | 0 |
| Northern Bobwhite | 0.4769 | 0.4679 | 0.469 | 0.4628 |
| Killdeer | 0.9537 | 0.9531 | 0.9526 | 0.9376 |
| Mourning Dove | 0.3114 | 0.3103 | 0.306 | 0.3015 |
| Northern Flicker | 0.5233 | 0.5188 | 0.2667 | 0 |
| Eastern Phoebe | 0.9994 | 0.9994 | 0.9864 | 0 |
| Eastern Kingbird | 0.9933 | 0.9939 | 0.9698 | 0 |
| Blue Jay | 0.8613 | 0.3835 | 0 | 0 |
| American Crow | 0.097 | 0.01 | 0 | 0 |
| Horned Lark | 0.9998 | 0.9998 | 0.9809 | 0 |
| Barn Swallow | 0.9999 | 0.9999 | 0.9999 | 0.9999 |
| Black-capped Chickadee | 0.9979 | 0.9977 | 0.9832 | 0 |
| House Wren | 0.9994 | 0.9995 | 0.9994 | 0 |
| Eastern Bluebird | 0.9998 | 0.9998 | 0.9998 | 0.8452 |
| American Robin | 0.9316 | 0.9316 | 0.9314 | 0.0313 |
| Cedar Waxwing | 0.7947 | 0.7995 | 0.8007 | 0.7481 |
| Common Yellowthroat | 0.9999 | 0.9999 | 0.9995 | 0 |
| Yellow Warbler | 0.9999 | 0.9998 | 0 | 0 |
| Chipping Sparrow | 0.996 | 0.9969 | 0.9774 | 0 |
| Field Sparrow | 0.9986 | 0.999 | 0.8249 | 0 |
| Vesper Sparrow | 0.9983 | 0.998 | 0.9979 | 0 |
| Savannah Sparrow | 0.9996 | 0.9996 | 0.9996 | 0 |
| Grasshopper Sparrow | 0.9978 | 0.9978 | 0.9933 | 0 |
| Northern Cardinal | 0.9672 | 0.9684 | 0.9691 | 0.4809 |
| Dickcissel | 0.9947 | 0.9931 | 0.9922 | 0 |
| Red-winged Blackbird | 0.9944 | 0.9943 | 0.9836 | 0 |
| Eastern Meadowlark | 0.9999 | 0.9999 | 0.9994 | 0 |
| Western Meadowlark | 0.9402 | 0.9379 | 0.9284 | 0 |
| Common Grackle | 0.8675 | 0.773 | 0 | 0 |
| American Goldfinch | 0.981 | 0.9805 | 0.9829 | 0.9814 |
| House Sparrow | 0.8466 | 0.8451 | 0.849 | 0.6909 |

Table G. Predicted mortality for 31 species associated with exposure to Permethrin by date of first pesticide application.

| Species | May | June | July | August |
| --- | --- | --- | --- | --- |
| Canada Goose | 0 | 0 | 0 | 0 |
| Northern Bobwhite | 0 | 0 | 0 | 0 |
| Killdeer | 0 | 0 | 0 | 0 |
| Mourning Dove | 0 | 0 | 0 | 0 |
| Northern Flicker | 0 | 0 | 0 | 0 |
| Eastern Phoebe | 0 | 0 | 0 | 0 |
| Eastern Kingbird | 0 | 0 | 0 | 0 |
| Blue Jay | 0 | 0 | 0 | 0 |
| American Crow | 0 | 0 | 0 | 0 |
| Horned Lark | 0 | 0 | 0 | 0 |
| Barn Swallow | 0 | 0 | 0 | 0 |
| Black-capped Chickadee | 0 | 0 | 0 | 0 |
| House Wren | 0 | 0 | 0 | 0 |
| Eastern Bluebird | 0 | 0 | 0 | 0 |
| American Robin | 0 | 0 | 0 | 0 |
| Cedar Waxwing | 0 | 0 | 0 | 0 |
| Common Yellowthroat | 0 | 0 | 0 | 0 |
| Yellow Warbler | 0 | 0 | 0 | 0 |
| Chipping Sparrow | 0 | 0 | 0 | 0 |
| Field Sparrow | 0 | 0 | 0 | 0 |
| Vesper Sparrow | 0 | 0 | 0 | 0 |
| Savannah Sparrow | 0 | 0 | 0 | 0 |
| Grasshopper Sparrow | 0 | 0 | 0 | 0 |
| Northern Cardinal | 0 | 0 | 0 | 0 |
| Dickcissel | 0 | 0 | 0 | 0 |
| Red-winged Blackbird | 0 | 0 | 0 | 0 |
| Eastern Meadowlark | 0 | 0 | 0 | 0 |
| Western Meadowlark | 0 | 0 | 0 | 0 |
| Common Grackle | 0 | 0 | 0 | 0 |
| American Goldfinch | 0 | 0 | 0 | 0 |
| House Sparrow | 0 | 0 | 0 | 0 |

Table H. Predicted fecundity (fledglings per female) for all 31 species and percent reductions associated with exposure to Carbaryl by date of first pesticide application.

| Species | Control Fecundity | % Reduct. May | % Reduct. June | % Reduct. July | % Reduct. August |
| --- | --- | --- | --- | --- | --- |
| Canada goose | 1.6 | 0 | 0 | 0 | 0 |
| Northern bobwhite | 2.3 | 0 | 0 | 0 | 0 |
| Killdeer | 0.8 | 5 | 5 | 3 | 4 |
| Mourning dove | 4.9 | 0 | 0 | 0 | 0 |
| Northern flicker | 4.0 | 0 | 0 | 0 | 0 |
| Eastern phoebe | 5.7 | 30 | 11 | 0 | 0 |
| Eastern kingbird | 1.4 | 12 | 4 | 0 | 2 |
| Blue Jay | 1.7 | 0 | 0 | 0 | 0 |
| American crow | 1.5 | 0 | 1 | 3 | 1 |
| Horned Lark | 6.1 | 7 | 3 | 0 | 0 |
| Barn swallow | 4.8 | 36 | 32 | 22 | 6 |
| Black-capped chickadee | 4.7 | 6 | 3 | 0 | 0 |
| House wren | 7.5 | 33 | 33 | 12 | 0 |
| Eastern bluebird | 6.3 | 9 | 7 | 1 | 0 |
| American robin | 5.2 | 0 | 0 | 0 | 0 |
| Cedar waxwing | 5.4 | 0 | 0 | 0 | 0 |
| Common yellowthroat | 4.3 | 40 | 30 | 1 | 0 |
| Yellow warbler | 2.4 | 93 | 20 | 0 | 0 |
| Chipping sparrow | 3.2 | 14 | 11 | 0 | 0 |
| Field sparrow | 3.0 | 52 | 21 | 0 | 0 |
| Vesper sparrow | 2.1 | 14 | 12 | 2 | 1 |
| Savannah sparrow | 2.7 | 31 | 35 | 9 | 1 |
| Grasshopper sparrow | 3.5 | 34 | 24 | 2 | 1 |
| Northern cardinal | 2.0 | 0 | 1 | 0 | 0 |
| Dickcissel | 1.7 | 10 | 8 | 1 | 0 |
| Red-winged blackbird | 1.2 | 2 | 0 | 0 | 0 |
| Eastern meadowlark | 3.4 | 12 | 7 | 0 | 1 |
| Western meadowlark | 3.2 | 0 | 0 | 0 | 0 |
| Common grackle | 2.9 | 0 | 0 | 0 | 0 |
| American goldfinch | 4.4 | 1 | 17 | 18 | 10 |
| House sparrow | 7.1 | 0 | 0 | 0 | 0 |

Table I. Predicted fecundity (fledglings per female) for all 31 species and percent reductions associated with exposure to Chlorpyrifos by date of first pesticide application.

| Species | Control Fecundity | % Reduct. May | % Reduct. June | % Reduct. July | % Reduct. Aug. |
| --- | --- | --- | --- | --- | --- |
| Canada goose | 1.6 | 100 | 98 | 0 | 0 |
| Northern bobwhite | 2.3 | 85 | 89 | 60 | 40 |
| Killdeer | 0.8 | 79 | 59 | 36 | 12 |
| Mourning dove | 4.9 | 54 | 42 | 30 | 17 |
| Northern flicker | 4.0 | 100 | 54 | 4 | 0 |
| Eastern phoebe | 5.7 | 68 | 45 | 1 | 1 |
| Eastern kingbird | 1.4 | 100 | 100 | 14 | 0 |
| Blue Jay | 1.7 | 45 | 0 | 0 | 0 |
| American crow | 1.5 | 18 | 1 | 3 | 1 |
| Horned Lark | 6.1 | 58 | 29 | 3 | 1 |
| Barn swallow | 4.8 | 100 | 64 | 47 | 16 |
| Black-capped chickadee | 4.7 | 100 | 21 | 3 | 1 |
| House wren | 7.5 | 100 | 73 | 42 | 0 |
| Eastern bluebird | 6.3 | 57 | 48 | 12 | 0 |
| American robin | 5.2 | 74 | 47 | 22 | 0 |
| Cedar waxwing | 5.4 | 99 | 100 | 59 | 17 |
| Common yellowthroat | 4.3 | 100 | 57 | 13 | 0 |
| Yellow warbler | 2.4 | 100 | 33 | 0 | 0 |
| Chipping sparrow | 3.2 | 100 | 53 | 13 | 0 |
| Field sparrow | 3.0 | 100 | 50 | 0 | 0 |
| Vesper sparrow | 2.1 | 100 | 62 | 25 | 0 |
| Savannah sparrow | 2.7 | 100 | 100 | 34 | 0 |
| Grasshopper sparrow | 3.5 | 100 | 59 | 17 | 0 |
| Northern cardinal | 2.0 | 75 | 49 | 25 | 0 |
| Dickcissel | 1.7 | 100 | 77 | 27 | 0 |
| Red-winged blackbird | 1.2 | 100 | 47 | 15 | 0 |
| Eastern meadowlark | 3.4 | 69 | 40 | 10 | 2 |
| Western meadowlark | 3.2 | 100 | 49 | 20 | 0 |
| Common grackle | 2.9 | 50 | 14 | 0 | 0 |
| American goldfinch | 4.4 | 99 | 100 | 100 | 50 |
| House sparrow | 7.1 | 69 | 48 | 28 | 7 |

Table J. Predicted fecundity (fledglings per female) for all 31 species and percent reductions associated with exposure to Indoxacarb by date of first pesticide application.

| Species | Control Fecundity | % Reduct. May | % Reduct. June | % Reduct. July | % Reduct. Aug. |
| --- | --- | --- | --- | --- | --- |
| Canada goose | 1.6 | 0 | 0 | 0 | 0 |
| Northern bobwhite | 2.3 | 0 | 1 | 0 | 0 |
| Killdeer | 0.8 | 16 | 19 | 16 | 3 |
| Mourning dove | 4.9 | 0 | 0 | 0 | 0 |
| Northern flicker | 4.0 | 0 | 0 | 0 | 0 |
| Eastern phoebe | 5.7 | 47 | 21 | 0 | 0 |
| Eastern kingbird | 1.4 | 36 | 29 | 0 | 0 |
| Blue Jay | 1.7 | 0 | 0 | 0 | 0 |
| American crow | 1.5 | 0 | 0 | 1 | 2 |
| Horned Lark | 6.1 | 18 | 15 | 0 | 0 |
| Barn swallow | 4.8 | 60 | 44 | 33 | 7 |
| Black-capped chickadee | 4.7 | 4 | 2 | 0 | 0 |
| House wren | 7.5 | 44 | 36 | 8 | 0 |
| Eastern bluebird | 6.3 | 21 | 34 | 4 | 0 |
| American robin | 5.2 | 3 | 2 | 0 | 0 |
| Cedar waxwing | 5.4 | 0 | 0 | 0 | 0 |
| Common yellowthroat | 4.3 | 40 | 26 | 0 | 0 |
| Yellow warbler | 2.4 | 97 | 7 | 1 | 1 |
| Chipping sparrow | 3.2 | 3 | 3 | 0 | 0 |
| Field sparrow | 3.0 | 18 | 11 | 0 | 0 |
| Vesper sparrow | 2.1 | 34 | 35 | 7 | 0 |
| Savannah sparrow | 2.7 | 56 | 62 | 16 | 1 |
| Grasshopper sparrow | 3.5 | 51 | 45 | 3 | 0 |
| Northern cardinal | 2.0 | 0 | 0 | 0 | 0 |
| Dickcissel | 1.7 | 34 | 39 | 13 | 0 |
| Red-winged blackbird | 1.2 | 3 | 7 | 0 | 0 |
| Eastern meadowlark | 3.4 | 17 | 24 | 0 | 1 |
| Western meadowlark | 3.2 | 2 | 7 | 0 | 0 |
| Common grackle | 2.9 | 0 | 0 | 0 | 0 |
| American goldfinch | 4.4 | 0 | 0 | 0 | 0 |
| House sparrow | 7.1 | 0 | 0 | 0 | 0 |

Table K. Predicted fecundity (fledglings per female) for all 31 species and percent reductions associated with exposure to λ-Cyhalothrin by date of first pesticide application.

| Species | Control Fecundity | % Reduct. May | % Reduct. June | % Reduct. July | % Reduct. Aug. |
| --- | --- | --- | --- | --- | --- |
| Canada goose | 1.6 | 0 | 0 | 0 | 0 |
| Northern bobwhite | 2.3 | 100 | 68 | 42 | 18 |
| Killdeer | 0.8 | 65 | 43 | 23 | 3 |
| Mourning dove | 4.9 | 53 | 37 | 20 | 3 |
| Northern flicker | 4.0 | 48 | 5 | 0 | 0 |
| Eastern phoebe | 5.7 | 55 | 35 | 0 | 0 |
| Eastern kingbird | 1.4 | 100 | 74 | 1 | 1 |
| Blue Jay | 1.7 | 6 | 0 | 0 | 0 |
| American crow | 1.5 | 1 | 1 | 2 | 2 |
| Horned Lark | 6.1 | 50 | 23 | 0 | 0 |
| Barn swallow | 4.8 | 100 | 60 | 41 | 10 |
| Black-capped chickadee | 4.7 | 39 | 8 | 0 | 1 |
| House wren | 7.5 | 100 | 52 | 21 | 0 |
| Eastern bluebird | 6.3 | 54 | 46 | 7 | 0 |
| American robin | 5.2 | 61 | 32 | 4 | 0 |
| Cedar waxwing | 5.4 | 100 | 67 | 28 | 0 |
| Common yellowthroat | 4.3 | 93 | 44 | 2 | 0 |
| Yellow warbler | 2.4 | 100 | 12 | 0 | 1 |
| Chipping sparrow | 3.2 | 74 | 37 | 1 | 0 |
| Field sparrow | 3.0 | 99 | 39 | 0 | 1 |
| Vesper sparrow | 2.1 | 93 | 50 | 13 | 0 |
| Savannah sparrow | 2.7 | 100 | 67 | 16 | 0 |
| Grasshopper sparrow | 3.5 | 96 | 48 | 4 | 0 |
| Northern cardinal | 2.0 | 62 | 34 | 10 | 0 |
| Dickcissel | 1.7 | 100 | 55 | 13 | 0 |
| Red-winged blackbird | 1.2 | 73 | 32 | 2 | 0 |
| Eastern meadowlark | 3.4 | 60 | 35 | 4 | 0 |
| Western meadowlark | 3.2 | 60 | 35 | 3 | 0 |
| Common grackle | 2.9 | 22 | 0 | 0 | 0 |
| American goldfinch | 4.4 | 100 | 100 | 76 | 34 |
| House sparrow | 7.1 | 58 | 35 | 15 | 0 |

Table L. Predicted fecundity (fledglings per female) for all 31 species and percent reductions associated with exposure to Malathion by date of first pesticide application.

| Species | Control Fecundity | % Reduct. May | % Reduct. June | % Reduct. July | % Reduct. Aug. |
| --- | --- | --- | --- | --- | --- |
| Canada goose | 1.6 | 96 | 95 | 0 | 0 |
| Northern bobwhite | 2.3 | 78 | 83 | 59 | 40 |
| Killdeer | 0.8 | 79 | 57 | 35 | 15 |
| Mourning dove | 4.9 | 49 | 38 | 28 | 18 |
| Northern flicker | 4.0 | 92 | 56 | 3 | 0 |
| Eastern phoebe | 5.7 | 67 | 45 | 1 | 1 |
| Eastern kingbird | 1.4 | 99 | 100 | 13 | 1 |
| Blue Jay | 1.7 | 45 | 2 | 0 | 0 |
| American crow | 1.5 | 28 | 1 | 2 | 1 |
| Horned Lark | 6.1 | 58 | 30 | 2 | 0 |
| Barn swallow | 4.8 | 100 | 64 | 48 | 16 |
| Black-capped chickadee | 4.7 | 100 | 22 | 3 | 1 |
| House wren | 7.5 | 100 | 73 | 42 | 0 |
| Eastern bluebird | 6.3 | 58 | 48 | 12 | 0 |
| American robin | 5.2 | 67 | 43 | 22 | 0 |
| Cedar waxwing | 5.4 | 99 | 99 | 58 | 18 |
| Common yellowthroat | 4.3 | 100 | 56 | 13 | 0 |
| Yellow warbler | 2.4 | 100 | 35 | 0 | 0 |
| Chipping sparrow | 3.2 | 100 | 53 | 12 | 0 |
| Field sparrow | 3.0 | 100 | 50 | 0 | 0 |
| Vesper sparrow | 2.1 | 100 | 62 | 24 | 0 |
| Savannah sparrow | 2.7 | 100 | 100 | 34 | 1 |
| Grasshopper sparrow | 3.5 | 100 | 60 | 17 | 0 |
| Northern cardinal | 2.0 | 76 | 50 | 23 | 0 |
| Dickcissel | 1.7 | 100 | 77 | 27 | 0 |
| Red-winged blackbird | 1.2 | 100 | 47 | 16 | 0 |
| Eastern meadowlark | 3.4 | 68 | 41 | 10 | 0 |
| Western meadowlark | 3.2 | 100 | 49 | 20 | 0 |
| Common grackle | 2.9 | 52 | 15 | 0 | 1 |
| American goldfinch | 4.4 | 100 | 100 | 100 | 50 |
| House sparrow | 7.1 | 67 | 47 | 27 | 6 |

Table M. Predicted fecundity (fledglings per female) for all 31 species and percent reductions associated with exposure to Methomyl by date of first pesticide application.

| Species | Control Fecundity | % Reduct. May | % Reduct. June | % Reduct. July | % Reduct. Aug. |
| --- | --- | --- | --- | --- | --- |
| Canada goose | 1.6 | 99 | 96 | 0 | 0 |
| Northern bobwhite | 2.3 | 56 | 66 | 49 | 36 |
| Killdeer | 0.8 | 77 | 57 | 35 | 13 |
| Mourning dove | 4.9 | 33 | 27 | 21 | 12 |
| Northern flicker | 4.0 | 88 | 38 | 1 | 0 |
| Eastern phoebe | 5.7 | 68 | 44 | 0 | 0 |
| Eastern kingbird | 1.4 | 100 | 100 | 13 | 2 |
| Blue Jay | 1.7 | 39 | 0 | 0 | 0 |
| American crow | 1.5 | 19 | 2 | 1 | 1 |
| Horned Lark | 6.1 | 58 | 30 | 3 | 0 |
| Barn swallow | 4.8 | 100 | 65 | 47 | 16 |
| Black-capped chickadee | 4.7 | 100 | 22 | 2 | 0 |
| House wren | 7.5 | 100 | 71 | 41 | 0 |
| Eastern bluebird | 6.3 | 58 | 48 | 11 | 0 |
| American robin | 5.2 | 72 | 47 | 21 | 0 |
| Cedar waxwing | 5.4 | 82 | 91 | 57 | 17 |
| Common yellowthroat | 4.3 | 100 | 57 | 12 | 0 |
| Yellow warbler | 2.4 | 100 | 33 | 0 | 0 |
| Chipping sparrow | 3.2 | 100 | 52 | 11 | 0 |
| Field sparrow | 3.0 | 100 | 50 | 0 | 0 |
| Vesper sparrow | 2.1 | 100 | 61 | 23 | 2 |
| Savannah sparrow | 2.7 | 100 | 100 | 34 | 0 |
| Grasshopper sparrow | 3.5 | 100 | 59 | 17 | 1 |
| Northern cardinal | 2.0 | 75 | 50 | 24 | 0 |
| Dickcissel | 1.7 | 100 | 78 | 29 | 0 |
| Red-winged blackbird | 1.2 | 100 | 49 | 16 | 0 |
| Eastern meadowlark | 3.4 | 69 | 41 | 11 | 1 |
| Western meadowlark | 3.2 | 97 | 50 | 21 | 0 |
| Common grackle | 2.9 | 47 | 12 | 0 | 0 |
| American goldfinch | 4.4 | 98 | 99 | 100 | 49 |
| House sparrow | 7.1 | 66 | 45 | 26 | 5 |

Table N. Predicted fecundity (fledglings per female) for all 31 species and percent reductions associated with exposure to Permethrin by date of first pesticide application.

| Species | Control Fecundity | % Reduct. May | % Reduct. June | % Reduct. July | % Reduct. Aug. |
| --- | --- | --- | --- | --- | --- |
| Canada goose | 1.6 | 7 | 0 | 0 | 0 |
| Northern bobwhite | 2.3 | 29 | 42 | 38 | 27 |
| Killdeer | 0.8 | 64 | 51 | 29 | 8 |
| Mourning dove | 4.9 | 19 | 20 | 19 | 6 |
| Northern flicker | 4.0 | 66 | 11 | 0 | 0 |
| Eastern phoebe | 5.7 | 60 | 42 | 0 | 1 |
| Eastern kingbird | 1.4 | 99 | 93 | 4 | 1 |
| Blue Jay | 1.7 | 16 | 0 | 0 | 0 |
| American crow | 1.5 | 0 | 0 | 2 | 3 |
| Horned Lark | 6.1 | 54 | 27 | 1 | 0 |
| Barn swallow | 4.8 | 67 | 62 | 46 | 13 |
| Black-capped chickadee | 4.7 | 66 | 14 | 1 | 1 |
| House wren | 7.5 | 90 | 58 | 35 | 0 |
| Eastern bluebird | 6.3 | 55 | 47 | 9 | 0 |
| American robin | 5.2 | 38 | 35 | 9 | 0 |
| Cedar waxwing | 5.4 | 30 | 50 | 41 | 3 |
| Common yellowthroat | 4.3 | 95 | 52 | 6 | 0 |
| Yellow warbler | 2.4 | 100 | 23 | 0 | 0 |
| Chipping sparrow | 3.2 | 84 | 46 | 6 | 0 |
| Field sparrow | 3.0 | 100 | 45 | 0 | 1 |
| Vesper sparrow | 2.1 | 95 | 57 | 19 | 2 |
| Savannah sparrow | 2.7 | 92 | 81 | 25 | 0 |
| Grasshopper sparrow | 3.5 | 96 | 54 | 11 | 1 |
| Northern cardinal | 2.0 | 42 | 39 | 14 | 0 |
| Dickcissel | 1.7 | 79 | 63 | 18 | 1 |
| Red-winged blackbird | 1.2 | 85 | 37 | 9 | 1 |
| Eastern meadowlark | 3.4 | 65 | 38 | 8 | 0 |
| Western meadowlark | 3.2 | 45 | 37 | 8 | 0 |
| Common grackle | 2.9 | 29 | 1 | 0 | 0 |
| American goldfinch | 4.4 | 29 | 71 | 91 | 44 |
| House sparrow | 7.1 | 33 | 33 | 17 | 0 |

Figure A. Estimated mortality due to exposure to each pesticide following a first application on 20 May.

Figure B. Percent reduction in fecundity due to exposure to each pesticide following a first application on 20 May.
